# Research progress and antibacterial mechanisms of plant essential oils as alternative therapies for periodontitis

**DOI:** 10.3389/fmicb.2026.1802802

**Published:** 2026-06-10

**Authors:** Yan Huang, Xiaoyi Liu, Li Chen, Maolin Li, Ying Xia, Kun Yang

**Affiliations:** Hospital/School of Stomatology, Zunyi Medical University, Zunyi, China

**Keywords:** antibacterial mechanisms, biofilm, delivery systems, inflammatory diseases, natural products

## Abstract

Periodontitis is a chronic inflammatory disease caused by subgingival plaque microorganisms, and its treatment is often limited by the antibiotic resistance of conventional drugs. Plant essential oils, owing to their wide availability, relatively high safety, and multi-target antibacterial properties, have emerged as potential alternative or adjunctive therapies for periodontitis. This review systematically summarizes recent research on the antibacterial activity and mechanisms of plant essential oils against the microbial complexes of subgingival plaque (red, orange, yellow, green, purple, and blue complexes). Findings indicate that various essential oils exhibit significant inhibitory effects against key pathogens (e.g., *P. gingivalis*, *T. forsythia*, *F. nucleatum*), with minimum inhibitory concentration values often below 100 μg/mL. Some essential oils effectively inhibit biofilm formation at sub-inhibitory concentrations. The primary mechanisms include disruption of bacterial cell membranes, interference with metabolic pathways, downregulation of virulence gene expression, and inhibition of quorum sensing systems. Specific individual components (e.g., carvacrol, thymol, eugenol, cinnamaldehyde, thymoquinone, and methyleugenol) demonstrate multi-target antibacterial properties, providing a basis for developing standardized oral care products. The application of delivery systems (e.g., nanoemulsions, liposomes) can enhance the stability and bioavailability of essential oils while preserving or augmenting their antibacterial activity. Despite their promising antibacterial potential, the practical application of plant essential oils is still constrained by issues such as low water solubility, residual toxicity, and chemical instability. Therefore, future efforts should focus on optimizing delivery systems and systematically evaluating their toxicological profiles. This review aims to provide a theoretical foundation for developing novel essential oil-based antibacterial agents against periodontitis and to offer new strategies for addressing the challenge of antibiotic resistance.

## Graphical abstract

**Figure:**
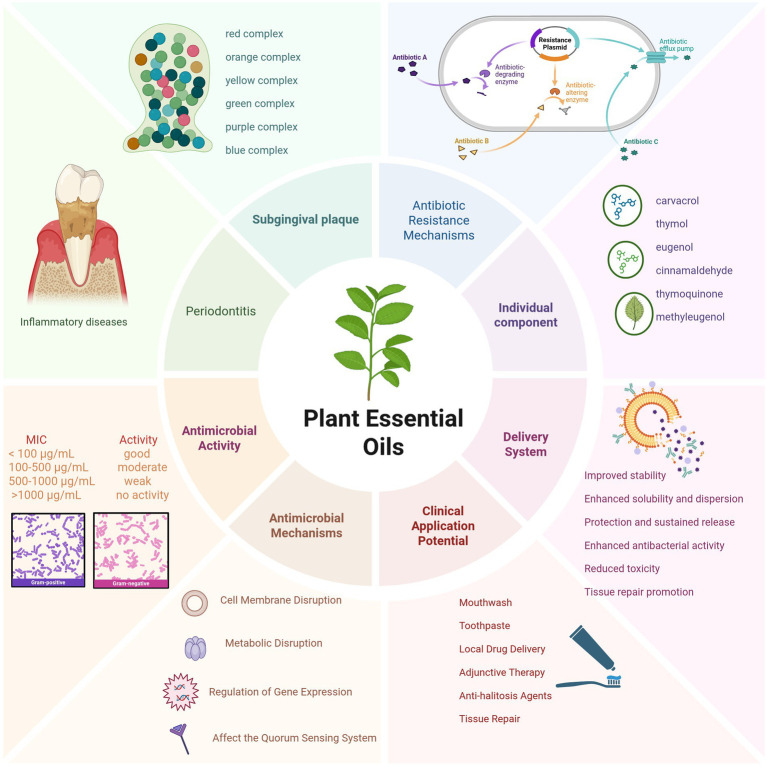

## 1 Introductions

**Figure 1:**
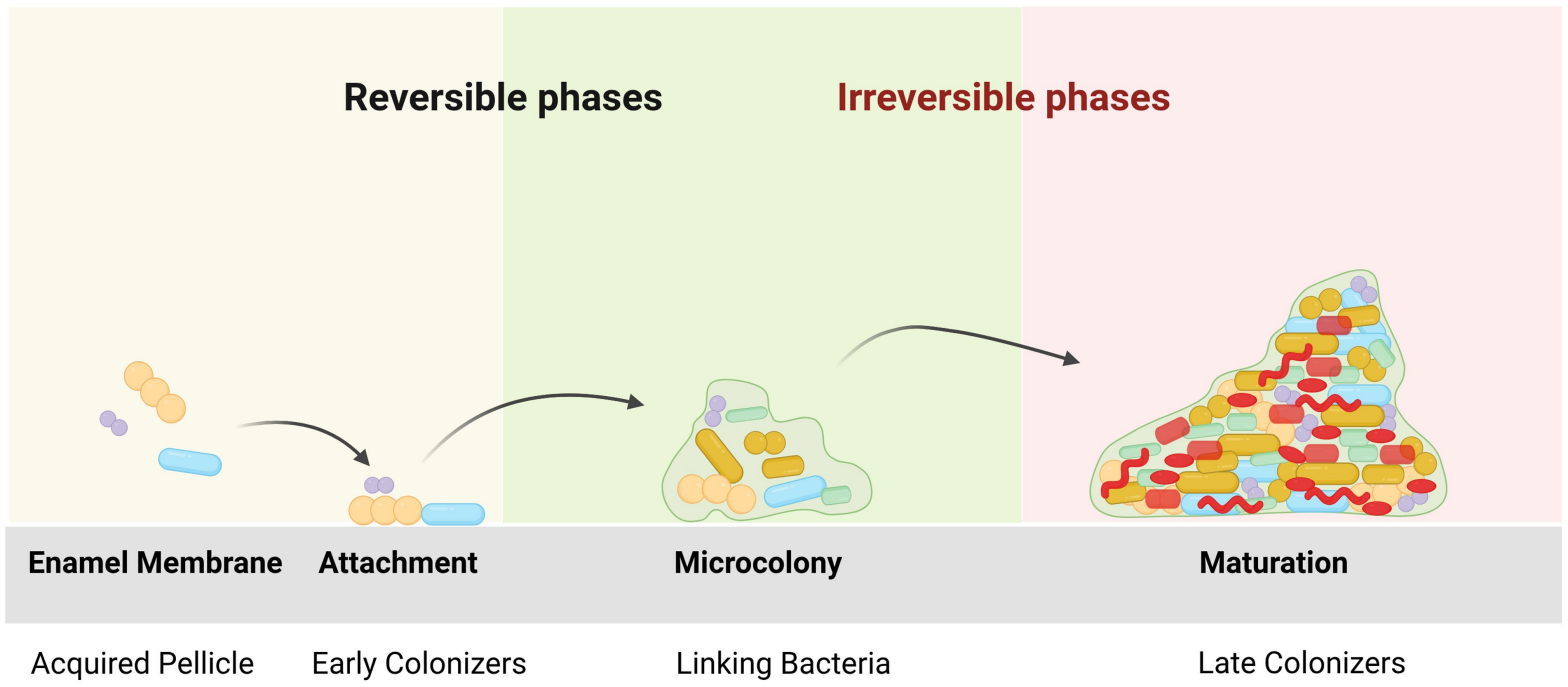
The process of biofilm formation. Source: Created with BioRender.com.

Periodontitis is a chronic inflammatory disease of the periodontal supporting tissues caused by dental plaque microorganisms. This condition is characterized by a high prevalence and a tendency to recur. Clinical manifestations primarily include inflammation of the periodontal tissues, progressive alveolar bone resorption, and tooth mobility, which may ultimately lead to tooth loss in severe cases (Kim et al., 2019). The oral microbiota is highly diverse and constitutes a complex ecosystem. Studies indicate that the oral cavity harbors over 700 species of microorganisms, including aerobes, facultative anaerobes, obligate anaerobes, and fungi, predominantly colonizing supragingival plaque, subgingival plaque, the dorsum of the tongue, and the buccal mucosa (Escapa et al., 2018). Subgingival plaque exhibits considerable complexity and diversity and is a primary etiological factor in periodontitis (Lamont et al., 2018). Based on clustering patterns and their association with periodontitis, Socransky et al. classified subgingival bacteria into six major microbial complexes, designated by colors: red, orange, yellow, green, purple, and blue. The “red complex” including *Porphyromonas gingivalis* (*P. gingivalis*), *Tannerella forsythia* (*T. forsythia*), and *Treponema denticola* (*T. denticola*), is closely linked to periodontitis. The “orange complex” including *Prevotella intermedia* (*P. intermedia*), *Prevotella nigrescens* (*P. nigrescens*), *Parvimonas micra* (*P. micra*; formerly *Peptostreptococcus micros*), and *Fusobacterium nucleatum* (*F. nucleatum*), facilitates the colonization and pathogenicity of the red complex. The “yellow complex” including *Streptococcus sanguinis* (*S. sanguinis*), *Streptococcus oralis* (*S. oralis*), *Streptococcus mitis* (*S. mitis*), *Streptococcus gordonii* (*S. gordonii*), and *Streptococcus intermedius* (*S. intermedius*), primarily comprises early colonizers involved in dental plaque biofilm formation. The “green complex” including *Aggregatibacter actinomycetemcomitans* (*A. actinomycetemcomitans*) and *Eikenella corrodens* (*E. corrodens*); the “purple complex” including *Veillonella parvula* (*V. parvula*); and the “blue complex” including *Actinomyces naeslundii* (*A. naeslundii*) and *Actinomyces viscosus* (*A. viscosus*) (Socransky et al., 1998). Figure 1 illustrates the process of biofilm formation.

Currently, the primary clinical approach for treating periodontitis involves the mechanical removal of dental calculus and plaque through supragingival scaling, subgingival scaling, and scaling and root planing (Saquib et al., 2019). Additionally, antibiotics such as metronidazole, tetracycline, azithromycin, and amoxicillin are often used as adjunctive therapies (Olsvik and Tenover, 1993; Pajukanta, 1993; Slots, 2002). However, microorganisms have developed various resistance mechanisms against conventional antibiotics, including: (1) utilizing efflux pumps to expel drugs and reduce intracellular concentrations; (2) altering drug targets to prevent binding and recognition; (3) secreting enzymes to degrade or inactivate drugs; (4) forming biofilms to impede drug penetration. These mechanisms collectively contribute to antibiotic failure, allowing bacterial proliferation and ultimately leading to treatment ineffectiveness (Cook and Wright, 2022; Upadhayay et al., 2023). Consequently, developing novel antimicrobial agents is crucial for mitigating bacterial resistance and improving clinical outcomes in infectious diseases.

Plants, being cost-effective, safe, and possessing broad biological activities, present a promising source for novel antimicrobial agents against periodontitis (Pasupuleti et al., 2023). Plant components are chemically diverse, with major active constituents including volatile oils, flavonoids, alkaloids, and organic acids (Qingjia et al., 2023; Fengping et al., 2019). Plant volatile oils, also known as essential oils, are volatile oily liquids produced as secondary metabolites, typically colorless, and found in various plant organs such as leaves, roots, bark, flowers, and fruits (Fengping et al., 2019). Chemically, essential oils are primarily composed of terpenes, terpenoids, phenylpropanoids, and other constituents. Numerous studies have demonstrated their antimicrobial properties (Pandey et al., 2016; Hyldgaard et al., 2012; Masyita et al., 2022). The antibacterial mechanisms of plant-derived antimicrobial agents include disrupting bacterial cell wall and membrane integrity, inhibiting protein and nucleic acid synthesis, and interfering with energy metabolism systems (Álvarez-Martínez et al., 2021). Compared to existing synthetic antibiotics, plant essential oils are natural complex mixtures containing dozens to hundreds of compounds. They can act on multiple bacterial targets simultaneously, such as the cell membrane and quorum sensing, making it difficult for bacteria to develop resistance. In contrast, synthetic antibiotics are single-component pure chemical substances that target only one specific site, readily inducing rapid resistance in bacteria through genetic mutations or enzymatic degradation. Furthermore, plant essential oils have a high safety profile, whereas synthetic antibiotics are often associated with systemic side effects such as gastrointestinal reactions or disruption of the normal microbiota (Sobhy et al., 2025; Mazumder et al., 2026).

This review summarizes recent research on the antibacterial activity and mechanisms of plant essential oils against subgingival plaque, aiming to provide a theoretical basis for the development of novel antibacterial agents for periodontitis. Although numerous studies have reported the antibacterial properties of plant essential oils, most existing reviews have focused primarily on single bacterial species or individual mechanisms of action, lacking a systematic analysis from the perspective of the overall structure of the subgingival plaque microecology. Furthermore, current research tends to emphasize *in vitro* antibacterial efficacy, while integrated elucidation of the multi-target antibacterial mechanisms of essential oils remains relatively limited. To address these gaps, this review systematically evaluates the differences in antibacterial activity of plant essential oils against the red, orange, yellow, green, purple, and blue microbial complexes of subgingival plaque, integrating their chemical composition characteristics. It further provides an in-depth analysis of their multi-target antibacterial mechanisms, including cell membrane disruption, metabolic interference, virulence gene regulation, and quorum sensing inhibition. In addition, this review incorporates recent advances in delivery systems that enhance the stability and bioavailability of essential oils. Compared with previous studies, this review constructs an analytical framework that spans “microbial complexes—active components—mechanisms of action—delivery systems,” thereby offering a more systematic theoretical foundation for the precise application of plant essential oils in periodontitis.

In this review, the reported antimicrobial concentrations (e.g., MIC, MBC, and DIZ values) are derived from the original cited studies, in which they were determined using standard microbiological methods such as broth microdilution, agar dilution, and disk diffusion assays. These values were extracted directly from the respective publications, with no further calculations performed beyond occasional necessary unit conversions (e.g., between μg/mL and mg/mL). It should be noted that variations in experimental conditions—including bacterial strain type (reference strain vs. clinical isolate), inoculum size, incubation conditions, solvent systems, and units of expression (e.g., μg/mL, μL/mL, % v/v)—may influence the reported values and limit direct cross-study comparability. Similarly, for plant essential oils, differences in extraction methods (e.g., steam distillation, hydrodistillation), GC–MS quantification approaches (e.g., peak area normalization for relative percentages vs. internal standard methods for absolute weight percentages), plant origin, and chemical composition may further contribute to variability in antimicrobial activity.

**Figure 2:**
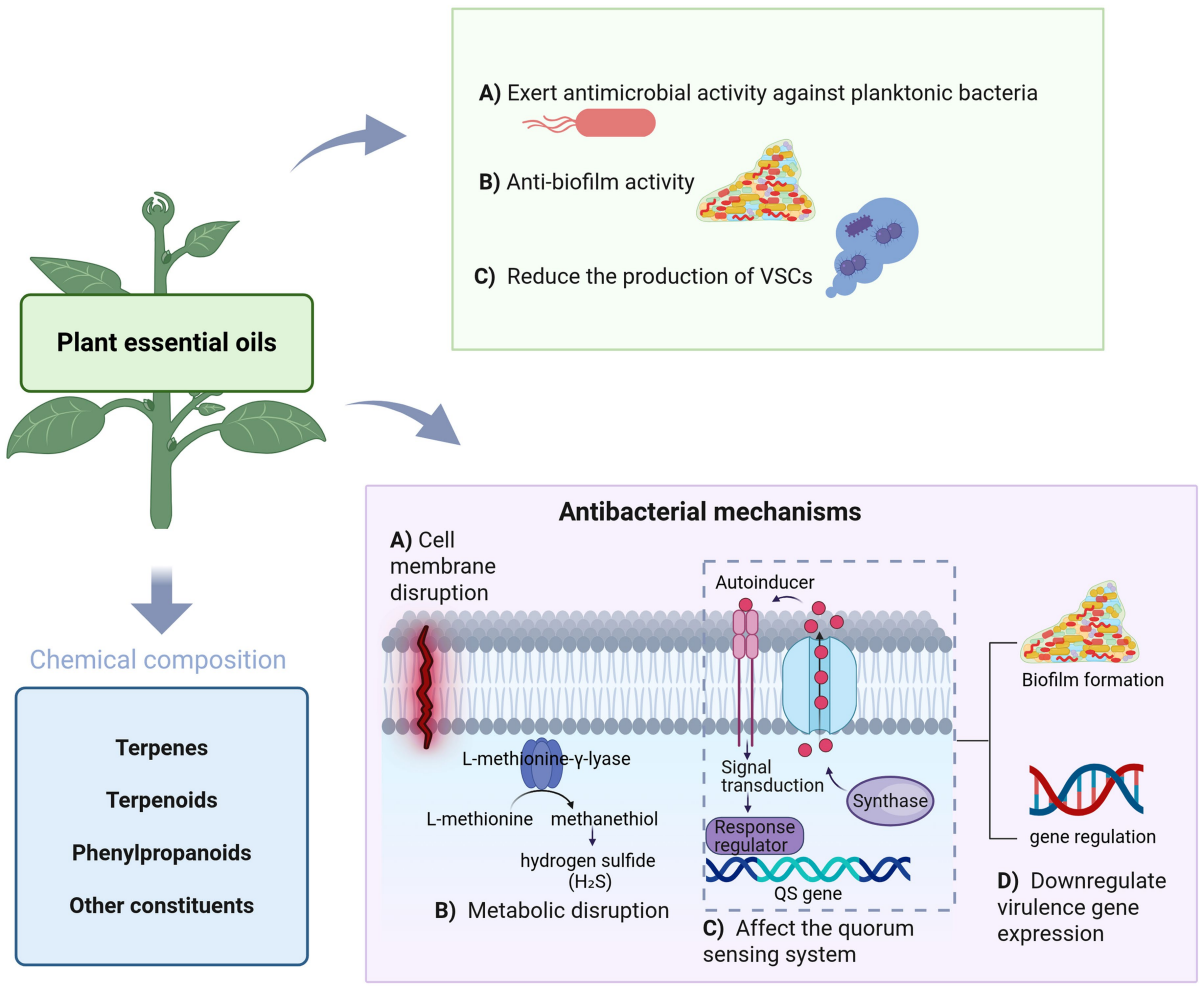
Overview of the antibacterial action of plant essential oils chemical constituents and core mechanisms. Source: Created with BioRender.com.

Figure 2 outlines the main chemical constituents of plant essential oils and illustrates their core antibacterial mechanisms-including cell membrane disruption, metabolic interference, gene expression regulation, and quorum sensing inhibition-thereby providing a systematic conceptual framework for this review.

## 2 Antimicrobial activity of plant essential oils

### 2.1 Antimicrobial activity against the red complex

#### 2.1.1 Antimicrobial activity against *Porphyromonas gingivalis*

The Gram-negative anaerobic bacterium *P. gingivalis* colonizes the subgingival region. It initiates infection by recognizing and adhering to host cells and extracellular matrix components through adhesins (Isolani et al., 2024). Gingipains are cytoplasmic-synthesized proteases secreted extracellularly. They function both as adhesive molecules mediating bacterial colonization and as proteolytic enzymes, contributing approximately 85% of *P. gingivalis* total proteolytic activity, thereby representing the most potent adhesins and virulence factors of this pathogen (Veillard et al., 2019).

Azeez et al. extracted essential oil from the gum of *Pistacia atlantica kurdica*, and its main constituent was *α*-pinene (79.76%). The essential oil exhibited both minimum inhibitory concentration (MIC) and minimum bactericidal concentration (MBC) of 12.5 μL/mL against *P. gingivalis* Clinical Isolate, indicating bactericidal potential (Azeez and Gaphor, 2019) [the ratio of MBC/MIC ≤ 4 suggests bactericidal activity, while the ratio > 4 indicates bacteriostatic activity (Pankey and Sabath, 2004)]. *Pistacia lentiscus* L. essential oil showed MIC values ranging from 1.63 to 3.13 μg/mL against reference strain *P. gingivalis* ATCC 33277 and two clinical isolates, demonstrating good antimicrobial activity (Milia et al., 2020) [according to established criteria: MIC < 100 μg/mL indicates good activity, 100–500 μg/mL moderate, 500–1000 μg/mL weak, and >1000 μg/mL no activity (Holetz et al., 2002)]. Notably, even within the same species (*Pistacia*), the chemical composition and antimicrobial efficacy of essential oils can vary depending on geographic origin. *Chrysopogon zizanioides* Roots essential oil demonstrated bactericidal potential against both reference strain *P. gingivalis* ATCC 33277 (MIC = 62.5 μg/mL, MBC = 150.0 μg/mL) and a clinical isolate (MIC = 100.0 μg/mL, MBC = 250.0 μg/mL) (Oliveira et al., 2022). Wongsariya et al. investigated the antimicrobial effects of *Citrus hystrix* leaves and peel essential oils against reference strain *P. gingivalis* ATCC 33277. The leaves oil showed weak activity (MIC = 1060 μg/mL, MBC = 1060 μg/mL), though theoretically bactericidal; however, the required effective concentration was high. Time-kill assays revealed complete lethality at 2 × MIC and 4 × MIC within 2 and 4 h, respectively, further supporting its potential bactericidal effect. The peel oil did not show significant antimicrobial activity (MIC>4620 μg/mL, MBC>4620 μg/mL) (Wongsariya et al., 2014). Hans et al. tested *Eucalyptus globulus*, *Melaleuca alternifolia*, *Matricaria chamomilla*, and *Curcuma longa* essential oils at 0, 25, 50, and 100% concentrations against a *P. gingivalis* clinical isolate using the disk diffusion method. At 25% concentration, diameters of inhibition zone (DIZ) for *Eucalyptus globulus* and *Matricaria chamomilla* were 1.70 ± 0.258 mm and 0.52 ± 0.199 mm, respectively, while *Melaleuca alternifolia* and *Curcuma longa* showed no significant activity. At 50%, they were: *Eucalyptus globulus* 2.51 ± 0.213 mm, *Melaleuca alternifolia* 0.98 ± 0.092 mm, *Matricaria chamomilla* 1.0 ± 0.2 mm, *Curcuma longa* 0.5 ± 0.082 mm. At 100%, they were: *Eucalyptus globulus* 4.5 ± 0.183 mm, *Melaleuca alternifolia* 2.9 ± 0.356 mm, *Matricaria chamomilla* 1.7 ± 0.183 mm, *Curcuma longa* 1.12 ± 0.079 mm. All four oils inhibited *P. gingivalis*, with larger inhibition zones observed at higher concentrations (Hans et al., 2016). Graziano et al. found that *Melaleuca alternifolia* essential oil inhibited *P. gingivalis* W83 at very low concentrations (MIC and MBC both 0.007%). At sub-MIC concentrations, it also reduced the production of volatile sulfur compounds (VSCs), potentially helping to mitigate oral malodor (Graziano et al., 2016). Mendoza et al. demonstrated that *Matricaria recutita* essential oil exhibits a certain degree of antibacterial activity against reference strain *P. gingivalis* ATCC 33277, with its inhibitory effect increasing in proportion to the concentration of the oil. After 24 h, DIZ were 15.55 ± 0.45 mm at 50% concentration and 18.90 ± 0.41 mm at 75%. After 48 h, they were 15.77 ± 0.46 mm and 19.22 ± 0.54 mm, respectively (Mendoza et al., 2023). *Salvia officinalis* and *Satureja kitaibelii* essential oils exhibited MIC values of 25 μg/mL and 12.5 μg/mL, respectively, against reference strain *P. gingivalis* ATCC 33277, indicating good activity, with *Satureja kitaibelii* showing stronger potency (Tambur et al., 2021). *Satureja montana* essential oil (MIC = 71.33 μg/mL, MBC = 142.66 μg/mL) demonstrated higher bactericidal potential against reference strain *P. gingivalis* ATCC 33277 than *Leptospermum scoparium* essential oil (MIC = 305.00 μg/mL, MBC = 1220.00 μg/mL). In a biofilm model formed on saliva-coated plates under anaerobic conditions for 24 h, both oils at their respective MICs significantly inhibited biofilm formation by over 85%, as shown by crystal violet staining (Yuan et al., 2025). *Syzygium aromaticum* essential oil showed bactericidal potential against reference strain *P. gingivalis* ATCC 33277 (MIC = 100 μg/mL, MBC = 100 μg/mL). The fractional inhibitory concentration (FIC) is defined as the ratio of the MIC of a single agent in combination to its MIC when used alone. The fractional inhibitory concentration index (FICI), calculated as the sum of the FICs of two combined agents, is used to classify drug interactions: synergy (FICI ≤ 0.5), additive effect (0.5 < FICI ≤ 1.0), indifference (1.0 < FICI ≤ 2.0), or antagonism (FICI > 2.0). In tests against the aforementioned microorganism using the checkerboard method, *Syzygium aromaticum* essential oil in combination with antibiotics exhibited synergistic effects, with FICI values ranging from 0.375 to 0.5. Time-kill assays further confirmed this synergy, showing an accelerated rate of bacterial killing when *Syzygium aromaticum* essential oil was combined with antibiotics (Moon et al., 2011). These findings provide new insights for clinical application. In periodontitis treatment, combining plant essential oils with antibiotics could significantly enhance bacterial killing efficiency, potentially allow for reduced antibiotic dosages while maintaining efficacy, help mitigate antibiotic resistance risks, and offer a potential natural-agent-based strategy for managing stubborn periodontal infections.

#### 2.1.2 Antimicrobial activity against *Tannerella forsythia*

*Tannerella forsythiais* is a key pathogen in the pathogenesis of periodontitis. Through its unique KLIKK protease/potempin inhibitor system, it attacks host tissues and evades immune clearance while simultaneously protecting its own outer membrane integrity, thereby playing a critical role in the initiation and progression of the disease (Książek et al., 2023).

*Pistacia lentiscus* L. essential oil demonstrated good antimicrobial activity against reference strain *T. forsythia* ATCC 43330 and two clinical isolates (Be 13,237, Be 13,216), with MIC values ranging from 1.63 to 3.13 μg/mL. Furthermore, this essential oil also exhibited good antimicrobial activity against reference strain *F. nucleatum* ATCC 25586, *S. gordonii* ATCC 10558, and *A. naeslundii* ATCC 12104, with corresponding MIC values of 6.25 μg/mL, 12.5 μg/mL, and 3.13 μg/mL (Milia et al., 2020). *Nigella sativa* seeds essential oil exhibited bactericidal potential against *T. forsythia* (DIZ = 13.05 ± 1.76 mm, MIC<31.2 μg/mL, MBC = 15.6 μg/mL). The oil also demonstrated activity against *P. gingivalis* (DIZ = 12.41 ± 0.41 mm, MIC = 31.2 μg/mL, MBC = 15.6 μg/mL), *P. intermedia* (DIZ = 12.12 ± 0.61 mm, MIC = 31.2 μg/mL, MBC = 15.6 μg/mL), and *A. actinomycetemcomitans* (DIZ = 15.11 ± 0.15 mm, MIC <31.2 μg/mL, MBC = 15.6 μg/mL) (Bhavikatti et al., 2024).

#### 2.1.3 Antimicrobial activity against *Treponema denticola*

*Treponema denticola* predominantly colonizes the subgingival area and is commonly found in the normal oral microbiota, where it can transform into a key opportunistic pathogen under conditions of microbial dysbiosis. The bacterium exerts multiple pathogenic effects through its surface chymotrypsin-like protease (CTLP). This protease serves not only as a critical virulence factor that facilitates tissue invasion and degradation of the basement membrane but also functions as an adhesin, mediating coaggregation with other pathogens such as *P. gingivalis* and promoting the formation of synergistic biofilms, thereby collectively exacerbating the destruction of periodontal tissues (Grenier et al., 1990; Cogoni et al., 2012).

Shapiro et al. evaluated the antibacterial activity of *Mentha piperita*, *Rosmarinus officinalis*, *Salvia officinalis*, *Ocimum tenuiflorum*, and *Australian Melaleuca alternifolia* essential oils against *T. denticola* strain CD-1. Only *Mentha piperita* and *Salvia officinalis* essential oils showed certain antimicrobial activity, with MIC values of 0.10% (w/v) and 0.20% (w/v), respectively; the remaining essential oils were not tested against this bacterium. Furthermore, the study found that all five essential oils exhibited varying degrees of antibacterial activity against *P. gingivalis*, *P. intermedia*, *P. nigrescens*, *F. nucleatum*, *S. sanguinis*, *A. actinomycetemcomitans*、*A. viscosus* (Shapiro et al., 1994).

In summary, as a core pathogenic microbial community in the development of periodontitis, the members of the red complex—*P. gingivalis*, *T. forsythia*, and *T. denticola*—work synergistically within periodontal tissues through multiple virulence factors to destroy host tissues and evade immune clearance. For instance, *P. gingivalis* primarily relies on gingipains and fimbrial adhesins to mediate colonization and proteolytic activity; *T. forsythia* employs its unique KLIKK protease/potempin inhibitor system to participate in tissue destruction while maintaining outer membrane stability; and *T. denticola* depends on the CTLP for tissue invasion, basement membrane degradation, and bacterial coaggregation. Collectively, these virulence factors promote the formation of synergistic biofilms and exacerbate the destruction of periodontal tissues. In terms of antimicrobial strategies, several plant essential oils have shown potential against red complex species. Essential oils such as those from *Pistacia atlantica kurdica*, *Pistacia lentiscus L.*, and *Chrysopogon zizanioides* Roots exhibit bactericidal potential against *P. gingivalis*, with most MIC values below 100 μg/mL, meeting the criteria for good antimicrobial activity. Oils from *Eucalyptus globulus*, *Melaleuca alternifolia*, *Matricaria chamomilla*, and *Curcuma longa* also inhibit the growth of this bacterium in a concentration-dependent manner. Certain essential oils, such as *Syzygium aromaticum* oil, demonstrate synergistic effects when combined with antibiotics, enhancing bactericidal efficiency and potentially reducing antibiotic dosage, thereby offering a new approach to mitigating resistance. *Pistacia lentiscus* L. and *Nigella sativa* seeds essential oils show promising antimicrobial activity against *T. forsythia*, while *Mentha piperita* and *Salvia officinalis* essential oils exhibit certain antibacterial effects against *T. denticola*. It is worth noting that the composition of essential oils can vary with geographical origin, which may lead to differences in their antimicrobial efficacy.

### 2.2 Antimicrobial activity against the orange complex

#### 2.2.1 Antimicrobial activity against *Prevotella* spp.

The Gram-negative anaerobic genus *Prevotella* spp. constitutes significant opportunistic pathogens in periodontitis, frequently co-existing with other pathogenic bacteria within periodontal pockets. These bacteria contribute to biofilm formation, host tissue destruction, and immune evasion, and they synergize with red complex bacteria to exacerbate periodontal inflammation and breakdown. Among the various species, *P. intermedia* and *P. nigrescens* are the most commonly implicated pathogenic types in periodontitis (Sharma et al., 2022; Albaghdadi et al., 2021).

*Chrysopogon zizanioides* Roots essential oil exhibited effective antimicrobial activity against both *P. intermedia* and *P. nigrescens* (MIC values ranged from 22.0 to 150.0 μg/mL, MBC values ranged from 62.5 to 400.0 μg/mL). Moreover, the essential oil also exhibited effective antimicrobial activity against *F. nucleatum* and *A. actinomycetemcomitans* (MIC values ranged from 22.0 to 250.0 μg/mL, MBC values ranged from 22.0 to 400.0 μg/mL) (Oliveira et al., 2022). In addition to evaluating the activity of *Matricaria recutita* essential oil against *P. gingivalis*, Mendoza et al. also investigated its effect on reference strain *P. intermedia* ATCC 25611. At 24 h, the DIZ for *P. intermedia* were 10.27 ± 0.18 mm at 50% concentration and 15.88 ± 0.29 mm at 75% concentration. At 48 h, the DIZ measured 10.49 ± 0.17 mm and 16.1 ± 0.33 mm, respectively. Notably, *P. intermedia* demonstrated lower sensitivity to *Matricaria recutita* essential oil compared to *P. gingivalis* (Mendoza et al., 2023). Essential oils extracted from four different parts (leaves, inner bark, outer bark, and wood) of *Kielmeyera coriacea* Mart. & Zucc. exhibited differences in their chemical compositions. The MIC values against reference strain *P. nigrescens* ATCC 33563 were 200 μg/mL for leaves, 50 μg/mL for inner bark, 100 μg/mL for outer bark, and 200 μg/mL for wood. The inner bark essential oil exhibited the strongest antibacterial activity, while the other three showed moderate activity (Martins et al., 2015). Essential oil of *Stachys koelzii* demonstrated bactericidal activity against reference strain *P. intermedia* ATCC 49046 (MIC = 100 μg/mL, MBC = 200 μg/mL). When *P. intermedia* was cultured in 96-well plates for 48 h to form biofilms, crystal violet staining and OD₅₇₀ measurement showed that, compared with the control, the biofilm biomass of *P. intermedia* was significantly reduced (OD₅₇₀ ≈ 0.1) after treatment with *Stachys koelzii* essential oil, indicating clear anti-biofilm activity. Moreover, the essential oil induced pronounced cytoplasmic leakage in *P. intermedia*, which increased over time (Ramak and Talei, 2018). Essential oils from *Satureja hortensis* Linnaeus, *Salvia fruticosa* Miller, *Lavandula stoechas* Linnaeus, *Myrtus communis* Linnaeus, and *Juniperus communis* Linnaeus all showed good antibacterial activity against *P. intermedia* and *P. nigrescens*. Among them, *Satureja hortensis* Linnaeus essential oil displayed strong activity (MIC < 0.125 μL/mL). Its main component was carvacrol (86.77%), a monoterpenoid phenol that disrupts the bacterial outer membrane, promotes lipopolysaccharide (LPS) release, and alters membrane permeability. In a biofilm model with periodontal pathogens cultured for 5 days in microtiter plates, at a sub-inhibitory concentration (0.01 μL/mL), only *Satureja hortensis* Linnaeus essential oil exhibited significant anti-biofilm activity against *P. nigrescens* AHN 8293, reducing the OD₅₉₅ to approximately 0.137 (Gursoy et al., 2009). Popa et al. also identified carvacrol (54.069%) as the main component of *Satureja hortensis* Linnaeus essential oil. This oil showed stronger antibacterial activity against oral *Prevotella* spp. (MIC = 680 μg/mL) than *Anethum graveolens* essential oil (MIC = 1,420 μg/mL). However, a much higher concentration was required to eradicate biofilms (minimal concentration for biofilm eradication: MBEC = 10,910 μg/mL for *Satureja hortensis* Linnaeus versus 2,840 μg/mL for *Anethum graveolens*). *Salvia officinalis* essential oil did not show significant antibacterial activity against oral *Prevotella* spp. (Popa et al., 2020).

#### 2.2.2 Antimicrobial activity against *Parvimonas micra*

*Parvimonas micra* is a common anaerobic coccus in the oral cavity and plays a significant role in periodontitis (Suresh et al., 2025). *P. micra* enhances its virulence expression through interactions with key pathogens such as *P. gingivalis*, notably by significantly promoting the activity of gingipains, thereby exacerbating periodontal tissue destruction (Neilands et al., 2019). Additionally, it can synergize with species such as *F. nucleatum* to form biofilms, which strengthens its colonization capacity and pathogenicity in subgingival plaque and collectively drives the progression of periodontal disease (Horiuchi et al., 2020).

A study by Gursoy et al. found that essential oils from *Satureja hortensis* Linnaeus, *Salvia fruticosa* Miller, *Lavandula stoechas* Linnaeus, *Myrtus communis* Linnaeus, and *Juniperus communis* Linnaeus exhibited antibacterial activity against *P. micra* (The MIC values were all ≤ 8 μL/mL), as well as against *P. gingivalis*, *T. forsythia*, *F. nucleatum*, and *A. actinomycetemcomitans*. Among these, *Satureja hortensis* Linnaeus essential oil showed the strongest antibacterial activity (MIC < 0.125 μL/mL), while *Salvia fruticosa* Miller essential oil displayed relatively weaker activity (MIC = 8 μL/mL) (Gursoy et al., 2009).

#### 2.2.3 Antimicrobial activity against *Fusobacterium nucleatum*

*Fusobacterium nucleatum*, a Gram-negative obligate anaerobe, can mediate coaggregation with nearly all oral bacterial species and adhere to a variety of host cells, thus acting as a bridging organism in dental plaque biofilm formation. It secretes virulence factors and metabolites that damage periodontal supporting tissues, induces host immune responses, and promotes the onset and progression of periodontal disease (Lima et al., 2017; Umaña et al., 2019). *F. nucleatum* is capable of producing a large amount of VSCs and is often regarded as one of the representative pathogens associated with halitosis (Amou et al., 2014).

Bersan et al. reported that essential oils from 20 Brazilian medicinal plants all exhibited certain antibacterial activity against reference strain *F. nucleatum* ATCC 25586. Among them, *Coriandrum sativum* L. essential oil demonstrated the strongest antibacterial activity (MIC = 15 μg/mL, MBC = 125 μg/mL). *Cyperus articulatus* L. essential oil (MIC = 250 μg/mL, MBC = 250 μg/mL) inhibited *F. nucleatum* biofilm formation by 60.42% at a concentration of 250 μg/mL. These 20 essential oils also showed certain activity against reference strain *P. gingivalis* ATCC 33277 (Bersan et al., 2014). *Satureja montana* essential oil exhibited the strongest antibacterial activity against three reference strain *F. nucleatum* strains (ATCC 25586, ATCC 10953, and ATCC 49256), with MIC and MBC values ranging from 0.030 to 0.063% (v/v). Its main component was carvacrol (43.8%). *Satureja montana* essential oil significantly increased the membrane permeability of *F. nucleatum* ATCC 25586 within 5 min, while *Rhododendron groenlandicum* and *Mentha piperita* essential oils required 60 min to achieve a similar effect. Both *Mentha piperita* and *Satureja montana* essential oil significantly reduced the activity of *F. nucleatum* ATCC 25586 single-species biofilms, by 69.1 and 91.8%, respectively. *Rhododendron groenlandicum* essential oil did not show significant anti-biofilm activity. All three essential oils reduced the production of VSCs (inhibition rates: 24.3 ~ 37.8%) (Ben Lagha et al., 2020). *F. nucleatum* JCM 11024 was susceptible to *Asarum heterotropoides* var. Mandshuricum essential oil (MIC = 0.01% [v/v], MBC = 0.02% [v/v]), showing higher sensitivity than *P. intermedia* JCM 12248 (MIC = 0.04% [v/v], MBC = 0.08% [v/v]), but lower sensitivity than *P. gingivalis* JCM 12257 (MIC = 0.005% [v/v/], MBC = 0.005% [v/v]). *Asarum heterotropoides* var. mandshuricum essential oil, whose main component is methyl eugenol (45.95%), effectively inhibited *F. nucleatum*-induced alveolar bone resorption in a mouse model (Wang et al., 2018). Methyl eugenol can interfere with bacterial acyl-homoserine lactone signaling, thereby disrupting quorum sensing, which subsequently suppresses pigment production, reduces motility and swarming ability, decreases exopolysaccharide synthesis and biofilm formation (Sybiya Vasantha Packiavathy et al., 2012). *Lavandula angustifolia* essential oil showed certain antibacterial activity against *F. nucleatum* PK1594 (MIC = 4 μL/mL). At concentrations of MIC and 2 × MIC, this oil significantly reduced the production of VSCs and the occurrence of halitosis (Rosner et al., 2024).

In summary, the orange complex plays a critical “bridging” and “synergistic” role in the progression of periodontitis. *Prevotella* spp., common anaerobic bacteria in periodontal pockets, directly damage tissues by producing various virulence factors and synergistically intensify inflammation with bacteria from the red complex. Essential oils such as *Chrysopogon zizanioides* Roots, *Matricaria recutita*, *Stachys koelzii*, and *Satureja hortensis* Linnaeus exhibit varying degrees of antibacterial activity against *Prevotella* spp. Among these, *Satureja hortensis* Linnaeus essential oil-rich in carvacrol-shows particularly strong efficacy. It not only effectively kills bacteria but can also inhibit biofilm formation even at subinhibitory concentrations. The mechanism is suggested to involve disruption of the bacterial outer membrane and subsequent LPS release. It is noteworthy that essential oils extracted from different parts of *Kielmeyera coriacea* Mart. & Zucc.—including leaves, inner bark, outer bark, and wood-display distinct chemical compositions and antibacterial properties. *P. micra* and *F. nucleatum* are two other key members of the orange complex. The former enhances overall virulence expression through interactions with pathogens such as *P. gingivalis*, while the latter acts as a crucial “bridging bacterium” due to its unique adhesin FadA, which is vital for dental plaque biofilm formation and structural stability. *F. nucleatum* is also a major producer of VSCs associated with halitosis. Studies indicate that essential oils like *Satureja hortensis* Linnaeus are also effective against *P. micra*. Research on the antibacterial activity against *F. nucleatum* is more extensive: *Satureja montana* oil (high in carvacrol) rapidly disrupts its cell membrane; methyl eugenol, the main component of *Asarum heterotropoides* var. mandshuricum essential oil, has been shown to interfere with bacterial quorum sensing, thereby inhibiting biofilm formation. Furthermore, *Asarum heterotropoides* var. mandshuricum essential oil has provided preliminary animal evidence by inhibiting bone resorption in mouse models. Additionally, various essential oils-including *Rhododendron groenlandicum*, *Mentha piperita*, *Satureja montana*, and *Lavandula angustifolia—*not only exhibit antibacterial effects but also significantly reduce VSCs production.

### 2.3 Antimicrobial activity against the yellow complex

Oral bacteria belonging to the genus *Streptococcus*, as early colonizers, initiate biofilm formation by specifically adhering to host proteins (such as mucins) within the salivary acquired pellicle, enabling their colonization at various sites in the oral cavity (Abranches et al., 2018).

*Citrus hystrix* leaves essential oil exhibited weak activity against reference strain *S. sanguinis* ATCC 10556 (MIC = 2,120 μg/mL, MBC = 4,250 μg/mL), while the peel essential oil showed no significant antimicrobial effect (MIC > 4,620 μg/mL, MBC > 4,620 μg/mL). Time-kill assays further demonstrated that complete lethality against *S. sanguinis* was achieved within 4 h at both 2 × MIC and 4 × MIC concentrations of the leaves essential oil (Wongsariya et al., 2014). Essential oils from 20 Brazilian medicinal plants exhibited certain antibacterial activity against reference strain *S. sanguinis* ATCC 10556 and *S. mitis* ATCC 903. Among them, *Mikania glomerata* Spreng essential oil demonstrated the strongest activity against *S. sanguinis* (MIC = 62 μg/mL, MBC = 125 μg/mL), and inhibited its biofilm formation by 54.79% at 1000 μg/mL. *Coriandrum sativum* L. essential oil showed the strongest antibacterial activity against *S. mitis* (MIC = 62 μg/mL, MBC = 125 μg/mL), but exhibited only minimal anti-biofilm activity (1.50%) against *S. mitis* at 1000 μg/mL (Bersan et al., 2014). The MIC value of *Satureja hortensis* L. essential oil against *S. sanguinis* PTCC 1449 was 1.5625% (v/v). Across the tested concentrations (50, 25, 12.5, 6.25, 3.125, and 1.5625% [v/v]), the corresponding DIZ were 28.83 ± 1.89 mm, 22 ± 1 mm, 18.6 ± 0.57 mm, 17 ± 1 mm, and 11.3 ± 1.15 mm, respectively. The results demonstrate a clear concentration-dependent antibacterial effect, with the DIZ gradually decreasing as the oil concentration declines (Hagh et al., 2019). Fresh leaf of *Psidium guajava* L. essential oil exhibited moderate antibacterial activity against reference strain *S. sanguinis* ATCC 10556 and *S. mitis* ATCC 49452, with MIC values of 200 and 400 μg/mL, respectively (Silva et al., 2019). Essential oil extracted from dry-seasons *Scheelea phalerata* showed moderate antibacterial activity, with MIC values of 200 μg/mL for reference strain *S. sanguinis* ATCC 10556 and 400 μg/mL for *S. mitis* ATCC 49456. It also demonstrated consistent antimicrobial effects against several key periodontal pathogens, including reference strain *P. gingivalis* ATCC 33277, *F. nucleatum* ATCC 25586, *A. actinomycetemcomitans* ATCC 43717, and *A. naeslundii* ATCC 19039, all with an MIC of 400 μg/mL (Oliveira et al., 2020). Essential oils derived from *Piper marginatum*, *Piper callosum*, and *Peperomia pellucida* exhibited MIC values of 225, 1,000, and 250 μg/mL, respectively against reference strain *S. sanguinis* ATCC 10556, while the corresponding MICs against *S. mitis* ATCC 49456 were 75, 500, and 125 μg/mL. Among these, *Piper marginatum* essential oil demonstrated the strongest antibacterial activity (Carvalho et al., 2022). At concentrations of 2.5, 5, 10, and 20 μg/mL, the DIZ for *S. sanguinis* PTCC 1449 were 7.07 ± 0.98, 12.0 ± 0.00, 12.17 ± 0.15, and 13.0 ± 0.00 mm with oleo-gum-resin of *Ferula assa-foetida* essential oil, and 6.94 ± 0.11, 7.93 ± 0.30, 9.90 ± 0.2, and 11.94 ± 0.20 mm with seeds of *Ferula assa-foetida* essential oil, respectively. The results indicate a positive dose-dependent relationship between the concentration of both essential oils and the size of the inhibition zone. Furthermore, the oleo-gum-resin essential oil exhibited stronger antibacterial activity than the seeds (Daneshkazemi et al., 2019). Essential oil from *Citrus aurantifolia* leaves exhibited MIC values of 200 μg/mL against both reference strain *S. sanguinis* ATCC 10556 and *S. mitis* ATCC 49456, whereas the fruit peel essential oil showed a lower MIC of 100 μg/mL against the same strains. Both oils demonstrated moderate antibacterial activity, with the fruit peel oil being more potent than the leaves. The primary component in both essential oils was limonene, but its concentration differed significantly-32.7% in the leaves oil compared to 77.5% in the peel oil (Lemes et al., 2018). Limonene exhibits antimicrobial activity against a range of Gram-positive and Gram-negative bacteria. Its mechanism primarily involves disrupting bacterial membrane integrity and increasing permeability, resulting in the leakage of intracellular contents and subsequent cell death. More specifically, limonene can damage the LPS structure, alter outer membrane permeability, and at higher concentrations, induce membrane rupture and structural disintegration, ultimately leading to cellular death (Gupta et al., 2021). Essential oils of *Artemisia dracunculus*, *Pimpinella anisum*, and *Citrus medica* produced DIZ of 16, 15, and 15 mm, respectively, against *S. sanguinis* PTCC1449. However, their MIC values were 28,440, 1820, and 200,000 μg/mL, indicating very weak antibacterial activity (Mohammadzadeh et al., 2025). *Cymbopogon martinii* essential oil exhibited an MIC of 250 μg/mL against both *S. sanguinis* and *S. mitis*, while *Thymus zygis* essential oil showed an MIC of 1,000 μg/mL against the same strains. *Cymbopogon martinii* essential oil demonstrated stronger antibacterial activity compared to *Thymus zygis*. This activity may be attributed to their high content of oxygenated monoterpenes (95.49 and 56.67%, respectively). These lipophilic compounds are effective in disrupting bacterial biofilm structures and killing embedded bacteria. Treatment with *Cymbopogon martinii* essential oil at MIC and 2 × MIC reduced the biofilm biomass of *S. mitis* to approximately 70%. In contrast, treatment with *Thymus zygis* essential oil at 1/2 × MIC reduced the biofilm biomass to about 50% (Marinković et al., 2020). *Thymus vulgaris* essential oil exhibited DIZ ranging from 18 to 36 mm against reference strain *S. sanguinis* ATCC 10556, *S. mitis* ATCC 9811, and *S. gordonii* ATCC 10558, with a consistent MIC value of 1.32 μg/mL for all three strains. In contrast, *Hyptis spicigera* essential oil showed narrower DIZ of 11–19 mm and MIC values ranging from 2.61 to 10.54 μg/mL. The stronger antibacterial activity of *Thymus vulgaris* essential oil may be attributed to its major compounds, thymol (25.22%) and carvacrol (23.78%) (de Oliveira et al., 2021). The combined effect of carvacrol and thymol disrupts the stability of microbial cell membranes. Specifically, carvacrol acts by lowering the local pH and interfering with the respiratory chain. Observations using atomic force microscopy revealed distinct alterations in the cell surface structure of microorganisms following exposure to carvacrol. The synergistic action of these phenolic compounds significantly enhances cell membrane permeability, which constitutes the core mechanism of their antibacterial activity (Ultee et al., 2002; La Storia et al., 2011; Lambert et al., 2001). *Cymbopogon citratus* essential oil demonstrated limited antibacterial activity against the tested strains, with inhibition zones of 10, 19, and 10 mm against reference strain *S. sanguinis* ATCC 10556, *S. mitis* ATCC 9811, and *S. gordonii* ATCC 10558, respectively, and MIC values of 2,610, 2,610, and 1,320 μg/mL for the same strains (Oliveira et al., 2017). Amil et al. investigated the antibacterial activity of four essential oils from the Zingiberaceae family: *Curcuma aeruginosa*, *C. mangga*, *C. xanthorrhiza*, and *Kaempferia galanga*. Among these, *C. xanthorrhiza* essential oil exhibited relatively strong activity against reference strain *S. sanguinis* ATCC 10556 and *S. mitis* ATCC 49456, producing DIZ of 19.50 ± 2.22 mm and 15.04 ± 3.05 mm, respectively. In contrast, *K. galanga* essential oil showed weaker antibacterial effects, with DIZ of 8.56 ± 1.02 mm and 7.18 ± 0.42 mm against the same two bacterial strains (Amil et al., 2024). Essential oils extracted from *Nepeta Cataria* at three different growth stages-vegetative, floral budding, and full flowering-all exhibited comparable antibacterial activity against reference strain *S. sanguinis* ATCC 10556, with no significant differences observed in their MIC (1 μL/mL) and MBC (2 μL/mL) values (Zomorodian et al., 2013). *Cimbopogon winterianus* essential oil showed weaker antibacterial activity against *S. intermedius* Clinical Isolate (MIC = 2.5% [v/v]) compared to *Origanum syriacum* essential oil (MIC = 1.25% [v/v]). Time-kill assays further confirmed that both oils inhibited the bacteria within 1–2 h; however, neither exhibited significant anti-biofilm activity (Rammal et al., 2024). Essential oil from *Tunisian Nigella sativa* seeds produced DIZ ranging from 10.5 ± 0.707 mm to 15.5 ± 0.707 mm against *S. mitis*, *S. sanguinis*, and *S. oralis*. However, with MIC values between 2,130 and 8,500 μg/mL, the overall antibacterial activity of the crude oil was limited. In contrast, its isolated component thymoquinone (0.79%) showed significantly stronger activity against the same bacteria, yielding larger DIZ (12.5 ± 0.7 mm to 22 ± 1.4 mm) and lower MIC values (8–256 μg/mL) (Harzallah et al., 2011). *Nigella sativa* (*NS*), commonly referred to as black cumin or black seed, is most frequently described for its bioactive component thymoquinone (2-isopropyl-5-methylbenzo-1,4-quinone). Thymoquinone inhibits periodontitis-associated pathogens by disrupting bacterial membrane integrity and reducing the expression of virulence factors. Topical applications, such as gels, have been shown in both clinical and experimental studies to effectively improve periodontal health parameters, highlighting its natural, multitarget antimicrobial potential (Mekhemar et al., 2020). The essential oil of *Melampodium divaricatum* (Rich.) DC. exhibited an MIC of 18 μg/mL against reference strain *S. mitis* ATCC 49456, while its individual components-*β*-caryophyllene (56%), caryophyllene oxide (3.0%), and *α*-humulene (1.9%)-showed MICs of 200, >400, and >400 μg/mL, respectively. Against reference strain *S. sanguinis* ATCC 10556, the essential oil had an MIC of 300 μg/mL, whereas the three components all displayed MICs >400 μg/mL. The antibacterial activity of the essential oil against both *streptococcal* strains was significantly stronger than that of its isolated constituents, suggesting that the overall effect likely results from synergistic interactions among the components (Moreira et al., 2014). *Pistacia vera* L. essential oil exhibited MIC values of 512, 1,024, and 256 μg/mL against reference strain *S. sanguinis* ATCC 10556, *S. oralis* ATCC 10557, and *S. intermedius* ATCC 27335, respectively, with *S. intermedius* showing the highest sensitivity. The main component of the oil, *α*-pinene (91.5%), displayed MIC values of 256, 1,024, and 512 μg/mL against the same strains. Notably, the overall antibacterial activity of the essential oil appears to result from more than just *α*-pinene alone; other constituents may interact synergistically or antagonistically with α-pinene, thereby modulating the oil’s total antimicrobial effect (Magi et al., 2018). *Plectranthus neochilus* essential oil demonstrated stronger antibacterial activity against reference strain *S. mitis* ATCC 49456 (MIC = 31.3 μg/mL) than against reference strain *S. sanguinis* ATCC 10556 (MIC = 62.5 μg/mL). The individual components α-pinene (14.1%), *β*-pinene (7.1%), trans-caryophyllene (29.8%), and caryophyllene oxide (12.8%) each showed an MIC of 4,000 μg/mL against *S. mitis*, with the mixture of all four also yielding an MIC of 4,000 μg/mL. For *S. sanguinis*, the MIC values of these components were 4,000, >4,000, >4,000, and >4,000 μg/mL, respectively, and the mixture exhibited an MIC >4,000 μg/mL. In both cases, the MIC values of the individual compounds and their mixture were substantially higher than that of the essential oil itself. This indicates that these major components are not the primary contributors to the oil’s antibacterial effect. The potent activity of the essential oil is more likely attributable to synergistic or additive interactions among its complex constituents or to the strong antimicrobial effects of its minor components (Crevelin et al., 2015). The individual compounds camphor (18.91%), verbenone (11.32%), *α*-pinene (9.61%), myrcene (8.56%), eucalyptol (7.97%), and *β*-caryophyllene (5.10%) exhibited MIC values against reference strain *S. mitis* ATCC 49456 of 300, 300, 400, 400, 300, and 300 μg/mL, respectively, and against reference strain *S. sanguinis* ATCC 10556 of 400, 400, 400, 1,500, 400, and 400 μg/mL. Most of these isolated components showed moderate antibacterial activity. In contrast, *Rosmarinus officinalis* essential oil itself demonstrated MICs > 2000 μg/mL against both *S. mitis* and *S. sanguinis*, indicating no significant antibacterial effect. This lack of activity may be attributed to the complexity of the essential oil’s composition and potential antagonistic interactions among its constituents (Bernardes et al., 2010).

In summary, as early colonizers within dental plaque biofilms, the yellow complex plays a key role in shaping the oral microenvironment and enabling the subsequent colonization of pathogenic bacteria. Extensive research has evaluated the antibacterial potential of various plant essential oils, revealing considerable variability in their efficacy, which stems largely from their complex chemical composition and the interactions among constituents. While certain oils, such as *Thymus vulgaris* essential oils-rich in thymol and carvacrol-demonstrate notable activity with MICs as low as 1.32 μg/mL against several *streptococcal* species, many others exhibit only limited or weak antibacterial effects. Comparative studies between whole essential oils and their primary isolated components highlight several recurring patterns. In some cases, the whole oil shows markedly greater activity than its main constituents-as observed with *Melampodium divaricatum* (*Rich.*) *DC.* and *Plectranthus neochilus* essential oil-implying synergy among minor components or between compounds. In other instances, the oil performs similarly to or even more weakly than its dominant component-such as *Pistacia vera* L. essential oil relative to *α*-pinene-suggesting either additive or antagonistic interactions. Conversely, certain essential oils display minimal antibacterial activity on their own, while individual constituents like thymoquinone from *Tunisian Nigella sativa* seeds essential oil or several monoterpenes from *Rosmarinus officinalis* essential oil show moderate to good activity independently, indicating that antagonistic effects within the complex mixture may attenuate the potential of its active components. Overall, as multicomponent natural blends, the bioactivity of essential oils represents the net outcome of synergistic, additive, or antagonistic interactions among their diverse chemical constituents.

### 2.4 Antimicrobial activity against the green complex

#### 2.4.1 Antimicrobial activity against *Aggregatibacter actinomycetemcomitans*

*Aggregatibacter actinomycetemcomitans* possesses multiple virulence factors, including outer membrane proteins (OM), leukotoxin (Ltx), and LPS. Through these specific factors, the bacterium disrupts the integrity of the gingival epithelial barrier, evades host immune clearance, and invades deeper periodontal supportive tissues, ultimately leading to the destruction of the periodontal ligament and alveolar bone (Gholizadeh et al., 2017; Oscarsson et al., 2019).

*Thymus vulgaris* essential oil demonstrated good antibacterial activity against *A. actinomycetemcomitans* Clinical Isolate (MIC = 32 μg/mL, DIZ = 8.2 ± 0.4 mm) (Fani and Kohanteb, 2017). *Origanum vulgare* essential oil, which contains carvacrol (32.36%) as its main component, demonstrated good antibacterial activity against all tested clinical and reference strains of *A. actinomycetemcomitans*. The MIC values ranged from 0.05 to 1.51 μg/mL, and the MBC values ranged from 0.09 to 2.01 μg/mL. All MBC/MIC ratios were below 4, indicating that *Origanum vulgare* essential oil exhibits bactericidal effects against *A. actinomycetemcomitans*. The oil did not significantly neutralize LtxA. Therefore, it does not interfere with the LtxA-neutralizing activity of *Psidium guajava* leaves extract and can be used in combination with it (Akkaoui et al., 2020). *Melaleuca alternifolia* essential oil showed an MIC of 16,700 μg/mL against reference strain *A. actinomycetemcomitans* ATCC 29522. In time-kill assays, the oil at the MIC concentration completely inhibited bacterial growth (Oliveira et al., 2024). *Cryptomeria japonica* essential oil demonstrated bactericidal potential against multiple oral pathogens (reference strain): *A. actinomycetemcomitans* ATCC 43717 (MIC = 400 μg/mL, MBC = 800 μg/mL), *P. gingivalis* ATCC 33277 (MIC = 25 μg/mL, MBC = 50 μg/mL), *P. intermedia* ATCC 25611 (MIC = 50 μg/mL, MBC = 100 μg/mL), *F. nucleatum* ATCC 10953 (MIC = 50 μg/mL, MBC = 100 μg/mL), *S. sanguinis* ATCC 10556 (MIC = 100 μg/mL, MBC = 100 μg/mL), and *S. gordonii* ATCC 10558 (MIC = 25 μg/mL, MBC = 50 μg/mL) (Cha et al., 2007). *Psidium cattleianum* Sabine (Myrtaceae) fresh leaves essential oil demonstrated good antibacterial activity against reference strain *A. actinomycetemcomitans* ATCC 43717 (MIC = 6.25 μg/mL), reference strain *P. gingivalis* ATCC 33277 (MIC = 20 μg/mL), reference strain *P. nigrescens* ATCC 33563 (MIC = 62.5 μg/mL), reference strain *F. nucleatum* ATCC 25586 (MIC = 12.5 μg/mL), and reference strain *A. naeslundii* ATCC 19039 (MIC = 50 μg/mL). Among these, *A. actinomycetemcomitans* showed the highest sensitivity to this essential oil (Chrystal et al., 2020).

#### 2.4.2 Antimicrobial activity against *Eikenella corrodens*

The detection rate of *E. corrodens* is high in patients with chronic periodontitis. Within the complex subgingival microbial community, *E. corrodens* acts synergistically with periodontal pathogens such as *A. actinomycetemcomitans*, *P. intermedia*, and *P. nigrescens*, collectively promoting the destruction of periodontal tissues and disease progression (Alsherif et al., 2023). *E. corrodens* can weaken host immune defenses and accelerate tissue degradation; its secreted thiol-dependent haemolysin exhibits significantly enhanced activity in the anaerobic reducing environment of periodontal pockets, disrupting host cells such as erythrocytes and exacerbating tissue damage (Allaker et al., 1994).

*Mentha piperita* and *Salvia officinalis* essential oils exhibited certain antibacterial activity against *E. corrodens*, with MIC values of 0.20% (w/v) and 0.10% (w/v), respectively (Shapiro et al., 1994). The plant mixtures (essential oils of *Salvia officinalis*, *Mentha piperita*, *Lippia citriodora* and aqueous extract of *Matricaria chamomilla*, *Echinacea purpurea*) exhibited bactericidal activity against *E. corrodens* PTCC1391 (MIC = 25 μg/mL, MBC = 25 μg/mL). Compared to *A. viscosus* PTCC1202 (MIC = 0.8 μg/mL, MBC = 12 μg/mL) and *S. sanguinis* PTCC1449 (MIC = 0.4 μg/mL, MBC = 6 μg/mL), *E. corrodens* showed lower sensitivity to the plant mixtures. Additionally, a combination of *Salvia officinalis, Mentha piperita*, and *Lippia citriodora* essential oils demonstrated anti-biofilm activity against all three bacterial species (Fathi et al., 2021).

In summary, within the green complex, *A. actinomycetemcomitans* and *E. corrodens* each secrete virulence factors that drive periodontal destruction: the former disrupts the epithelial barrier and invades deeper tissues through Ltx and LPS, while the latter secretes thiol-dependent hemolysin to interfere with immune responses, synergistically exacerbating tissue damage. In terms of antimicrobial activity, *Origanum vulgare, Thymus vulgaris, and Psidium cattleianum* Sabine (Myrtaceae) fresh leaves essential oils exhibit good antibacterial effects against *A. actinomycetemcomitans*, with *Origanum vulgare* essential oil demonstrating the strongest activity. *Origanum vulgare* essential oil, primarily composed of carvacrol, exhibits bactericidal effects against all tested strains of *A. actinomycetemcomitans* and does not neutralize the activity of LtxA, thus allowing *Psidium guajava* leaves extract to maintain its inhibitory effect against this toxin, making it suitable for combination therapy. Meanwhile, the plant mixtures of *Salvia officinalis, Mentha piperita*, and *Lippia citriodora* essential oils demonstrated certain antibacterial and anti-biofilm activity against *E. corrodens.*

### 2.5 Antimicrobial activity against the purple complex

#### 2.5.1 Antimicrobial activity against *Veillonella parvula*

*Veillonella parvula* can coaggregate with *S. gordonii*. Although this coaggregation does not significantly alter bacterial gene expression, it determines the spatial architecture of dental plaque biofilms, promotes intimate interbacterial contact and metabolic cross-feeding, thereby providing a physical scaffold for subsequent colonizing bacteria and driving the oral microenvironment toward periodontitis progression (Dorison et al., 2024).

Both *Cinnamomum camphora cineoliferum* and *Melaleuca ericifolia* essential oils are rich in oxygenated monoterpenes, with contents of 68.70 and 80.00%, respectively. The *Cinnamomum camphora cineoliferum* essential oil (MIC = 4,550 μg/mL, MBC = 9,100 μg/mL) and *Melaleuca ericifolia* essential oil (MIC = 650 μg/mL, MBC = 1,350 μg/mL) both exhibited limited antibacterial activity against *V. parvula* (Marinkovic et al., 2022).

### 2.6 Antimicrobial activity against the blue complex

#### 2.6.1 Antimicrobial activity against *Actinomyces*

*Actinomyces* is one of the pioneer species in biofilm formation, capable of achieving initial adhesion and colonization on tooth surfaces, thereby providing a structural basis for subsequent microbial coaggregation (Li et al., 2004). *A. naeslundii*, which colonizes the innermost layer of the biofilm, modulates the local microenvironment-such as pH and oxygen levels-through its unique metabolic activities, creating favorable conditions for the subsequent colonization of more anaerobic pathogenic bacteria (Dige et al., 2009).

Essential oils from four different parts of *Kielmeyera coriacea* Mart. & Zucc.—leaves, inner bark, outer bark, and wood-were tested against reference strain *A. naeslundii* ATCC 19039. The MIC values obtained were >400 μg/mL for leaves, >400 μg/mL for inner bark, 400 μg/mL for outer bark, and >400 μg/mL for wood. Only the outer bark essential oil demonstrated moderate antibacterial activity, while other oils showed no detectable activity against this strain (Martins et al., 2015). Essential oils from *Satureja hortensis* Linnaeus, *Salvia officinalis*, and *Anethum graveolens* all demonstrated certain antibacterial activity against *A. naeslundii*. Among them, *Satureja hortensis* Linnaeus essential oil exhibited moderate antibacterial activity against *A. naeslundii* S 41.2 (MIC = 340 μg/mL) (Popa et al., 2020). Essential oils from *Thymus vulgaris* and *Hyptis spicigera* produced DIZ of 24 mm and 19 mm, respectively, against reference strain *A. naeslundii* ATCC 4356, with corresponding MIC values of 0.32 μg/mL and 1.32 μg/mL. *A. naeslundii* was more susceptible to *Thymus vulgaris* essential oil, which also demonstrated a certain interference effect during the early stages of biofilm development (de Oliveira et al., 2021). *Pimpinella anisum* essential oil exhibited certain antibacterial activity against *A. naeslundii* PTCC 1201 (DIZ = 42 ± 1.63 mm, MIC = 4.88% [v/v], MBC = 9.76% [v/v]). *A. naeslundii* showed higher susceptibility to *Pimpinella anisum* essential oil compared to *A. actinomycetemcomitans* JP2NOV99 (DIZ = 18.5 ± 1.29 mm, MIC = MBC = 9.76% [v/v]). This difference in sensitivity may be attributed to the structural distinction between Gram-positive and Gram-negative bacteria. Unlike Gram-positive bacteria, the outer membrane of Gram-negative bacteria contains a LPS layer that restricts penetration to only specific hydrophilic compounds (Bakhshi et al., 2022). Sabinene (36.6%) is the primary constituent of *Myristica fragrans* essential oil. The essential oil demonstrated good antibacterial activity against reference strain *A. viscosus* ATCC 10048 (MIC = 8 μg/mL), reference strain *P. gingivalis* ATCC 33277 (MIC = 8 μg/mL), and reference strain *P. intermedia* ATCC 25611 (MIC = 4 μg/mL), with *P. intermedia* exhibiting the highest sensitivity (Setty et al., 2020). Liu et al. extracted essential oil from *Lavandula angustifolia* using hydrodistillation (HD) and microwave-assisted hydrodistillation (MAHD). The results indicated that while the chemical composition of the oils obtained by the two methods was highly consistent, the content of individual compounds varied considerably. *Lavandula angustifolia* essential oil extracted by MAHD demonstrated stronger antibacterial activity against *A. viscosus* (DIZ = 10.2 ± 0.3 mm, MIC = 125 μg/mL) compared to that obtained by HD (DIZ = 9.3 ± 0.6 mm, MIC = 250 μg/mL), suggesting that MAHD offers distinct advantages over the conventional HD method (Liu et al., 2018).

In summary, bacteria of the *Actinomyces* within the blue complex adhere to and colonize tooth surfaces, thereby modulating the local microenvironment to facilitate the proliferation of subsequent anaerobic pathogens and promote the progression of periodontitis. Regarding the inhibition of these bacteria, various plant essential oils have demonstrated antibacterial effects at different levels, thus interfering with early biofilm formation. *Thymus vulgaris* essential oil exhibits good inhibitory activity against *A. naeslundii* (MIC = 0.32 μg/mL). *Pimpinella anisum* essential oil shows notable antibacterial effects; due to differences in cell envelope structure, *A. naeslundii* is more susceptible to *Pimpinella anisum* essential oil than *A. actinomycetemcomitans*. Furthermore, extraction methods influence the efficacy of essential oils, as illustrated by *Lavandula angustifolia* essential oil obtained via MAHD displaying superior antibacterial activity against *A. viscosus* compared to that extracted by HD. However, certain essential oils, such as that from the outer bark of *Kielmeyera coriacea* Mart. & Zucc., only show moderate activity, while essential oils from other parts of the plant exhibit no significant inhibition, indicating that the antibacterial effectiveness of essential oils varies with their botanical source and chemical composition.

## 3 Antimicrobial mechanisms of plant essential oils

### 3.1 Cell membrane disruption

Plant essential oils are complex mixtures of low molecular weight compounds, primarily existing as hydrocarbons or structures containing functional groups such as aldehydes, alcohols, esters, and ketones. Their lipophilic nature and low molecular weight enable them to diffuse and penetrate the bacterial outer membrane, irreversibly compromising its structural integrity. This disruption leads to an imbalance in membrane permeability and ultimately results in bacterial death (Sikkema et al., 1995; Filoche et al., 2005). Wongsariya et al. used scanning electron microscopy (SEM) to examine the effects of *Citrus hystrix* leaves essential oil at 4 × MIC on *P. gingivalis*. The degree of cell membrane damage in *P. gingivalis* increased with longer exposure time. After 8 h of treatment, complete disruption of the bacterial cell membrane was observed, ultimately leading to the leakage of intracellular contents (Wongsariya et al., 2014). Bersan et al. (2014) used SEM to observe that *Cyperus articulatus* L. essential oil induced alterations in cell membrane morphology in *P. gingivalis*, *F. nucleatum*, *S. sanguinis*, and *S. mitis*. Transmission electron microscopy (TEM) revealed that treatment with *Rhododendron groenlandicum*, *Mentha piperita*, and *Satureja montana* essential oils for 60 min induced membrane disruption in *F. nucleatum* (Ben Lagha et al., 2020). As the concentration of *Lavandula angustifolia* essential oil increased (0.5 × MIC, MIC, and 2 × MIC), the integrity of the *F. nucleatum* cell membrane was progressively disrupted, with damage rates reaching 30, 60, and 70%, respectively. Subsequent observation under fluorescence microscopy provided direct visual evidence of this membrane disruption (Rosner et al., 2024). The plant mixtures (essential oils of *Salvia officinalis*, *Mentha piperita*, *Lippia citriodora* and aqueous extract of *Matricaria chamomilla*, *Echinacea purpurea*) induced significant damage to the cell membrane of *S. sanguinis*. Transmission electron microscopy revealed bacterial membrane invagination, disruption, and severe alterations in the cell wall, accompanied by a morphological shift from the typical coccoid form to swollen, elliptical, and other abnormal shapes (Fathi et al., 2021). Zeidán-Chuliá et al. demonstrated through N-phenyl-1-naphthylamine (NPN) fluorescence assays that both *Satureja hortensis* L. and *Sal. fruticosa* M. essential oils significantly increased the outer membrane permeability of *F. nucleatum* (Zeidán-Chuliá et al., 2013). *Cinnamomum zeylanicum* bark essential oil induced the leakage of nucleic acids and proteins from *P. gingivalis* and increased the number of propidium iodide-positive bacterial cells, indicating disruption of the bacterial cell membrane and enhanced membrane permeability. Subsequently, SEM revealed that bacterial cells exposed to the essential oil displayed irregular morphology, pronounced wrinkling, surface depressions, and the formation of pores, further confirming irreversible damage to the cell membrane of *P. gingivalis* (Wang et al., *2018*). Li et al. observed via SEM that *P. gingivalis* and *F. nucleatum* treated with *Elsholtzia ciliate* essential oil lost their original morphology and exhibited significant rupture and damage. Subsequent propidium iodide staining further confirmed that the essential oil acts on the bacterial cell membrane, increasing its permeability and ultimately leading to membrane disruption and bacterial death (Li et al., 2023). Terpinen-4-ol is a major component of essential oils from plants such as *Melaleuca alternifolia*. SEM revealed that upon exposure to terpinen-4-ol, the cell membrane structures of *P. gingivalis*, *P. intermedia*, and *F. nucleatum* undergo significant alterations (Kamiya et al., 2024).

### 3.2 Metabolic disruption

Methanethiol, a volatile sulfur compound produced by *F. nucleatum* through the catalytic action of L-methionine-*γ*-lyase, represents one of the primary contributors to halitosis (Wu et al., 2020). *Nigella sativa* essential oil and its active constituent thymoquinone demonstrated MIC values of 63 μg/mL and 31 μg/mL, respectively. At a concentration of 10 μg/mL, *Nigella sativa* essential oil significantly inhibited the L-methionine-γ-lyase activity in *F. nucleatum*, leading to a marked reduction in the production of the metabolites *α*-ketobutyrate and ammonia. Further analysis revealed that thymoquinone, at 16.4 μg/mL, exhibited mixed-type inhibition against this enzyme. Since both effective concentrations were below their respective MIC values, these findings suggest that the mechanism involves the specific inhibition of the metabolic enzyme pathway in *F. nucleatum*, rather than direct bactericidal action (Ishikawa et al., 2021).

### 3.3 Regulation of gene expression

*Porphyromonas gingivalis* employs a range of surface structures and molecules to regulate its virulence, including fimbriae, LPS, capsules, proteases, and hemagglutinins (Lamont and Jenkinson, 1998). The *fimA* gene primarily encodes the major subunit (*FimA* protein) of *P. gingivalis* fimbriae, which is essential for biofilm formation, adherence to host cells, and interaction with the oral microbiota (Hasegawa and Nagano, 2021). *P. gingivalis* does not produce siderophores but instead relies on a coordinated set of genes to acquire and utilize iron and heme. The *ragA*, *rgpA*, *rgpB*, *kgp*, and *vimA* genes are associated with gingipain production; *hagA*, *hagB*, and *hagE* genes encode hemagglutinins; *hem* genes are involved in membrane disruption and erythrocyte lysis; *hmuR* participates in hemoglobin binding and degradation; and *ftn* encodes the iron-storage protein ferritin (Olczak et al., 2005).

*Syzygium aromaticum* leaves essential oil demonstrated bactericidal potential against *P. gingivalis* (MIC = 6.25 μg/mL, MBC = 25 μg/mL). RT-qPCR analysis confirmed that eugenol (90.84%), the primary component of *Syzygium aromaticum* leaves essential oil, significantly downregulated the expression of multiple virulence genes in *P. gingivalis*, including *fimA* (23.8%), *hagA* (76.7%), *hagB* (77.2%), *rgpA* (25.9%), *rgpB* (25.0%), and *kgp* (18.3%), thereby impairing its adhesion and tissue-destructive capacity (Zhang et al., 2017). As the primary component of *Cinnamomum zeylanicum* essential oil, cinnamaldehyde exhibited good antibacterial activity against *P. gingivalis* (MIC = 21.12 μg/mL). RT-qPCR analysis confirmed that cinnamaldehyde significantly influences the mRNA expression of multiple virulence genes, including *ragA*, *rgpA*, *rgpB*, *kgp*, *vimA*, *hagA*, *hagB*, *hagE*, *hem*, *ftn*, and *humR*, thereby further elucidating its inhibitory effect on *P. gingivalis* and the underlying mechanisms involved (Qiaoqiao, 2023). *Satureja montana* and *Leptospermum scoparium* essential oils were observed to downregulate the expression of key virulence factors in *P. gingivalis*, including *hagA*, *hagB*, *hem*, *hmuR*, *ragA*, *ftn*, and *fimA-I*. Molecular docking studies further demonstrated that the active constituents of *Satureja montana* essential oil (carvacrol, *γ*-terpinene, and p-cymene), as well as those of *Leptospermum scoparium* essential oil (leptospermone), exhibited strong binding affinity to multiple virulence-associated proteins. These findings suggest a multi-target antimicrobial mechanism underlying the activity of these essential oils (Yuan et al., 2025).

### 3.4 Affect the quorum sensing system

Quorum sensing (QS) is a communication mechanism employed by both Gram-positive and Gram-negative bacteria, involving the secretion and release of specific signaling molecules. By detecting changes in the concentration of these molecules, bacteria monitor population density and regulate physiological functions to adapt to their environment (Miller and Bassler, 2001). QS plays a crucial role in processes such as the release of virulence factors, biofilm formation, and adhesion (Yi et al., 2021). Studies have identified that plant-derived QS inhibitors (QSIs) primarily consist of terpenoids, flavonoids, and alkaloids (Damte et al., 2013; Adonizio et al., 2006). Molecular docking analysis revealed that linalool (48.17%), L-limonene (22.03%), and *α*-terpineol (7.31%)-the primary constituents of *Citrus bergamia* essential oil-can form stable interactions with several key regulatory proteins (3QP5, 5OE3, 4B2O, 3Q3D) involved in QS, indicating the potential of *Citrus bergamia* essential oil to interfere with bacterial QS. Further *in vitro* experiments confirmed that *Citrus bergamia* essential oil significantly suppresses bacterial biofilm formation and inhibits violacein production in the QS model bacterium *Chromobacterium violaceum*. These results suggest that *Citrus bergamia* essential oil may inhibit bacterial biofilm activity by disrupting QS pathways (Aziz et al., 2024).

Although studies specifically addressing the inhibition of QS in periodontal bacterial biofilms by plant essential oils are currently limited, the previously discussed essential oils have demonstrated anti-biofilm activity and the suppression of virulence factor expression. Notably, these oils often contain components such as linalool, limonene, and *α*-terpineol, which are associated with anti-QS properties. Therefore, it is plausible that plant essential oils may also possess the potential to inhibit QS in periodontal pathogens. Research conducted by our group has shown that *Houttuynia cordata* essential oil effectively reduces the biomass of single-species biofilms formed by *S. sanguinis*, *F. nucleatum*, and *P. gingivalis*, and disrupts their three-dimensional architecture. Furthermore, it was observed that this essential oil significantly decreases the activity of AI-2 signal molecules (as measured by luminescence) in the culture supernatants of these bacteria and downregulates the transcriptional level of the *LuxS* gene. These findings suggest that *H. cordata* essential oil may inhibit biofilm formation by interfering with the LuxS/AI-2 QS pathway.

## 4 Delivery system

Nowadays, plant essential oils are widely studied for their antimicrobial properties. However, their high volatility and poor water solubility limit practical application. To improve stability, researchers have begun developing delivery systems. Manconi et al. encapsulated *Thymus capitatus* essential oil into phospholipid vesicles, including liposomes, glycerosomes, and propylene glycol-containing vesicles (PG-PEVs). *In vitro* studies demonstrated that these vesicles exhibited a unilamellar spherical morphology, uniform size distribution, relatively high entrapment efficiency (approximately 47–51%), and good storage stability. Notably, formulations containing 12.5–25% glycerol or propylene glycol maintained nearly constant particle size over 60 days, showing significantly improved stability compared to conventional liposomes. These vesicular systems, by modulating the lipid bilayer structure and reducing interfacial tension, help minimize the volatility and degradation of the essential oil, thereby enhancing its protection and enabling sustained delivery. Further investigations revealed that the vesicles did not compromise the antibacterial activity of *Thymus capitatus* essential oil against cariogenic bacteria, while maintaining low aggressiveness toward commensal oral microbiota. Importantly, the formulations exhibited excellent biocompatibility in keratinocytes (cell viability ≥ 100%) and significantly promoted cell proliferation and migration by enhancing vesicle–cell membrane interactions and cellular uptake, thereby accelerating wound healing. These pro-regenerative effects are primarily attributed to the antioxidant properties of the essential oil and the improved intracellular delivery efficiency conferred by the vesicular carriers, rather than any cytotoxic effects (Manconi et al., 2018). Chen et al. microencapsulated cinnamaldehyde using CGTase-catalyzed products. The resulting emulsion exhibited stronger antibacterial activity than free cinnamaldehyde, primarily attributed to better aqueous dispersion, enhanced membrane permeability (evidenced by decreased pH, increased conductivity, and protein leakage), and more pronounced degradation of membrane proteins. Microscopy and flow cytometry further revealed that the emulsion induced more severe cell damage and a higher mortality rate. Moreover, the emulsion retained full antibacterial activity after two years of storage, demonstrating excellent stability (Chen et al., 2024).

Although plant essential oils (EOs) are naturally derived, their safety is not absolute, as they are chemical compounds produced by plants for self-protection and may exhibit toxic effects in humans. Such toxicity can be local or systemic and varies depending on the oil’s chemical composition, harvest season, ecotype, plant part used, and geographic origin. At the cellular level, essential oils from the *Mentha* spp. and their main constituents (e.g., menthol, limonene, and apiol) can induce mitochondrial dysfunction and disrupt cell membranes, exerting cytotoxic effects on human tumor cells and inhibiting cell proliferation. Systemic toxicity is also evident; for example, *Mentha pulegium*, which contains pulegone, is hepatotoxic, and mint oils may additionally cause nausea, allergic reactions, and interfere with drug metabolism, highlighting potential drug–oil interactions in clinical applications (Stringaro et al., 2018). To mitigate these toxic effects while enhancing bioactivity, recent studies have explored technological improvements. For instance, *Cinnamomum cassia* oil formulated as a nanoemulsion shows increased stability and bioavailability, with significantly stronger antibacterial activity against both Gram-positive and Gram-negative bacteria than the unmodified oil (Liang et al., 2022). Similarly, although high concentrations of *Melaleuca alternifolia* oil exhibit toxicity, combining it with chitosan reduces its toxicity and bacterial resistance, demonstrating a strong synergistic effect. These findings indicate that physical or chemical modifications of essential oils can effectively preserve their antimicrobial and biological activities while significantly improving safety (Oliveira et al., 2024).

## 5 Clinical application potential

The cornerstone of periodontitis treatment lies in the inhibition of plaque biofilm formation, making pharmacological control of dental plaque a crucial adjunctive approach. Among chemical agents, chlorhexidine is widely regarded as the gold standard for plaque control in dentistry. However, its use as a mouthwash is associated with side effects such as dry mouth, taste disturbance, tooth staining, and a potential risk of promoting calculus formation. Consequently, there is a growing need to develop mouthwashes with reduced synthetic chemical content and enhanced natural bioactive ingredients (Sharma et al., 2024). Most plant essential oils have demonstrated potent antimicrobial activity against subgingival microorganisms while also helping to reduce oral malodor. This positions them as promising candidates for the development of effective and natural mouthwashes aimed at controlling oral bacteria and treating halitosis (Graziano et al., 2016; Ishikawa et al., 2021). Furthermore, plant essential oils can be incorporated into toothpaste formulations or utilized in aromatherapy for the prevention and management of oral infections (Fani and Kohanteb, 2017). Studies have indicated that plant essential oils may promote the regeneration of oral fibroblasts without adversely affecting their viability and show no significant cytotoxicity toward oral cells. Coupled with their inherent antimicrobial properties, these oils hold potential as natural preservatives (Milia et al., 2020).

Among plant essential oils, certain individual components have demonstrated clear potential for application. Carvacrol [derived from essential oils such as *Satureja hortensis* Linnaeus (Gursoy et al., 2009) and *Origanum vulgare* (Akkaoui et al., 2020)] and thymol [obtained from *Thymus vulgaris* essential oil (de Oliveira et al., 2021)] exhibits activity in disrupting bacterial cell membranes. Thymoquinone [derived from the essential oil of *Tunisian Nigella sativa* seeds (Harzallah et al., 2011; Ishikawa et al., 2021)] not only inhibits metabolic enzymes in *F. nucleatum* and disrupts bacterial cell membranes but also shows potential to improve periodontal parameters in local applications. Eugenol [sourced from *Syzygium aromaticum* leaves essential oil (Zhang et al., 2017)] significantly downregulates the expression of virulence genes in *P. gingivalis*. Methyl eugenol [obtained from *Asarum heterotropoides* var. mandshuricum essential oil (Wang et al., 2018)] has been shown to interfere with bacterial QS and inhibit biofilm formation. Limonene [present in essential oils such as *Citrus aurantifolia* (Lemes et al., 2018) and *Citrus bergamia* (Aziz et al., 2024)] is also reported to damage bacterial cell membranes and exhibit potential QS inhibitory activity. These individual bioactive components offer more precise options for developing standardized oral care products based on plant essential oils-such as mouthwashes, toothpastes, topical gels, and antibacterial sprays-and provide a scientific foundation for translating natural extracts into targeted therapeutic agents.

## 6 Conclusion and future perspectives

Subgingival plaque plays a pivotal role in the initiation and progression of periodontal diseases. However, the growing challenge of antibiotic resistance has driven the need to develop novel antibacterial agents. Plant essential oils have emerged as a highly promising alternative therapy due to their potent, low-toxicity, and broad-spectrum antimicrobial activities. This review summarizes the inhibitory effects of plant essential oils on subgingival plaque, with most demonstrating favorable to moderate antibacterial efficacy. Analysis of the relationship between chemical composition and biological activity reveals distinct advantages among different classes of bioactive constituents: monoterpene phenols (carvacrol, thymol) within the terpene/terpenoid group exhibit membrane-disruptive properties; phenylpropanoids (eugenol, cinnamaldehyde, methyl eugenol) attenuate bacterial pathogenicity by downregulating virulence genes and interfering with quorum sensing; while other compounds such as thymoquinone not only disrupt bacterial cell membranes but also inhibit metabolic pathways associated with oral malodor. These findings offer new perspectives for clinical application: rational formulation of plant-derived monomers with complementary mechanisms of action, or their combination with low-dose antibiotics, may enhance therapeutic efficacy while delaying the development of resistance. Such approaches could provide improved strategies for managing refractory periodontal infections.

Nevertheless, plant essential oils still faces certain limitations. Their strong hydrophobicity and poor water solubility may restrict their dispersion and bioavailability within the oral environment. Some essential oils exhibit residual toxicity or irritation at higher concentrations, warranting further evaluation of their safety for oral mucosa. Moreover, the components of essential oils are susceptible to degradation induced by light, oxygen, and temperature fluctuations, leading to variability in their stability and therapeutic efficacy. Therefore, future research should focus on the development of delivery systems that enhance the solubility and stability of essential oils, along with systematic assessment of their toxicological profiles, thereby facilitating the safe and effective translation of plant essential oils into periodontal therapy.

Given the substantial compositional variation among essential oils from different botanical sources, future efforts should prioritize the development of standardized extraction and quality control methods. Notably, research on the antibacterial mechanisms of essential oils has largely focused on their ability to disrupt bacterial cell membranes-altering permeability and inducing leakage of cellular contents. However, essential oils likely exert their effects through multiple targets and pathways. Therefore, future studies should employ proteomics, metabolomics, and other systemic approaches to comprehensively elucidate how essential oils modulate bacterial metabolic networks and signaling pathways, thereby providing a holistic understanding of their antimicrobial mechanisms. Our research group has employed proteomic analysis and found that *Houttuynia cordata* essential oil broadly influences the expression of proteins associated with virulence, metabolism, stress response, and secretion systems in bacterial outer membrane vesicles. These findings suggest that this essential oil may exert antibacterial effects by interfering with multiple pathways, including intercellular communication, energy metabolism, and virulence factor secretion.
