# Supplementary material for: Research progress and antibacterial mechanisms of plant essential oils as alternative therapies for periodontitis
Source: Front Microbiol. 2026 Jun 10;17:1802802. doi: 10.3389/fmicb.2026.1802802 (PMC13290721; doi:10.3389/fmicb.2026.1802802)
Supplement: Supplementary file 1 [file Table_1.DOCX]

**Table 1.** A Summary of the Main Components of Plant Essential Oils and Their Antibacterial Efficacy Against Subgingival Periodontal Bacteria: MIC, MBC, bacteriostatic Zone, Anti-biofilm Activity, and Mechanisms.

| Essential oils | Main component | Bacteria | MIC | MBC | Bacteriostatic/Bactericidal activity | DIZ(mm ± S.D) | Anti-biofilm activity or Reduce VSCs production | Mechanism | Ref. |
| --- | --- | --- | --- | --- | --- | --- | --- | --- | --- |
| *Pistacia atlantica Kurdica* | alpha-Pinene (79.76%) | *P. gingivalis* Clinical Isolate | 12.5 μL/mL | 12.5 μL/mL | Bactericidal | - | - | - | [22] |
| *Pistacia lentiscus* L. | α-pinene (16.89±0.15%), terpinen-4-ol (16.49±0.18%), Sabinene (7.73±0.11%) | *P. gingivalis* ATCC 33277  *P. gingivalis* BeOR6  *P. gingivalis* BeOR14  *T. forsythia* ATCC 43330  *T. forsythia* Be13237  *T. forsythia* Be13216  *F. nucleatum* ATCC 25586  *S. gordonii* ATCC 10558  *A. naeslundii* ATCC 12104 | 3.13 µg/mL  1.63 µg/mL  1.63 µg/mL  3.13 µg/mL  1.63 µg/mL  3.13 µg/mL  6.25 µg/mL  12.5 µg/mL  3.13 µg/mL | -  -  -  -  -  -  -  -  - | -  -  -  -  -  -  -  -  - | -  -  -  -  -  -  -  -  - | -  -  -  -  -  -  -  -  - | -  -  -  -  -  -  -  -  - | [24] |
| *Chrysopogon zizanioides* Roots | khusimol (30.0 ± 0.3%), β-eudesmol (10.8 ± 0.3%), α-muurolene (6.0 ± 0.1%) | *P. gingivalis* ATCC 33277  *P. gingivalis* Clinical Isolate  *P. intermedia* ATCC 49046  *P. intermedia* Clinical Isolate  *P. nigrescens* ATCC 33563  *F. nucleatum* ATCC 25586  *F. nucleatum* Clinical Isolate  *A. actinomycetemcomitans* ATCC 43717 | 62.5 µg/mL  100 µg/mL  22 µg/mL  150 µg/mL  62.5 µg/mL  50 µg/mL  250 µg/mL  22 µg/mL | 150 µg/mL  250 µg/mL  400 µg/mL  400 µg/mL  62.5 µg/mL  50 µg/mL  400 µg/mL  22 µg/mL | Bactericidal  Bactericidal  bacteriostatic  Bactericidal  Bactericidal  Bactericidal  Bactericidal  Bactericidal | -  -  -  -  -  -  -  - | -  -  -  -  -  -  -  - | -  -  -  -  -  -  -  - | [26] |
| *Citrus hystrix* leaves | β-Citronellal (78.11%), Citronellyl acetate (6.24%), β-Citronellol (5.28%) | *P. gingivalis* ATCC 33277  *S. sanguinis* ATCC 10556 | 1060 µg/mL  2120 µg/mL | 1060 µg/mL  4250 µg/mL | Bactericidal  Bactericidal | -  - | -  - | Cell membrane disruption  - | [27] |
| *Citrus hystrix* peel | - | *P. gingivalis* ATCC 33277  *S. sanguinis* ATCC 10556 | ＞4620 µg/mL  ＞4620 µg/mL | ＞4620 µg/mL  ＞4620 µg/mL | -  - | -  - | -  - | -  - | [27] |
| *Eucalyptus globulus*  (Concentration: 0、25、50、100%) | - | *P. intermedia* Clinical Isolate | - | - | - | 0 (0%)  1.70 ± 0.258 (25%)  2.51 ± 0.213 (50%)  4.5 ± 0.183 (100%) | - | - | [28] |
| *Melaleuca alternifolia* (Concentration: 0、25、50、100%) | - | *P. intermedia* Clinical Isolate | - | - | - | 0 (0%)  0 (25%)  0.98 ± 0.092 (50%)  2.9 ± 0.356 (100%) | - | - | [28] |
| *Matricaria chamomilla*  (Concentration: 0、25、50、100%) | - | *P. intermedia* Clinical Isolate | - | - | - | 0 (0%)  0.52 ± 0.199 (25%)  1 ± 0.2 (50%)  1.7 ± 0.183 (100%) | - | - | [28] |
| *Curcuma longa*  (Concentration: 0、25、50、100%) | - | *P. intermedia* Clinical Isolate | - | - | - | 0 (0%)  0 (25%)  0.5 ± 0.082 (50%)  1.12 ± 0.079 (100%) | - | - | [28] |
| *Melaleuca alternifolia* | terpinen-4-ol (42.07%), gamma-terpinene (19.72%)  alpha-terpinene (9.13%) | *P. gingivalis* W83 | 0.007 % | 0.007 % | Bactericidal | - | Reduce VSCs production | - | [29] |
| *Matricaria recutita*  (Concentration: 50、75%) | - | *P. gingivalis* ATCC 33277  *P. intermedia* ATCC 25611 | -  - | -  - | -  - | 15.55 ± 0.45 (24hours、50%)  15.77 ± 0.46 (48hours、50%)  18.9 ± 0.41 (24hours、75%)  19.22 ± 0.54 (48hours、75%)  10.27 ± 0.18 (24hours、50%)  10.49 ± 0.17 (48hours、50%)  15.88 ± 0.29 (24hours、75%)  16.1 ± 0.33 (48hours、75%) | -  - | -  - | [30] |
| *Salvia officinalis* | - | *P. gingivalis* ATCC 33277 | 25 µg/mL | - | - | - | - | - | [31] |
| *Satureja kitaibelii* | - | *P. gingivalis* ATCC 33277 | 12.5 µg/mL | - | - | - | - | - | [31] |
| *Satureja montana* | γ-terpinene (20.20±0.38%), p-cymene (16.01±0.66%), carvacrol (14.50±0.35%) | *P. gingivalis* ATCC 33277 | 71.33 µg/mL | 142.66 µg/mL | Bactericidal | 42.06 ± 1.62 | Anti-biofilm activity | Downregulated the expression of *hagA, hagB, hem, hmuR, ragA, ftn,* and *fimA-I* gene | [32] |
| *Leptospermum scoparium* | leptospermone (17.44±0.40%), cis-muurola-4(14), 5-diene (10.55±0.24%) | *P. gingivalis* ATCC 33277 | 305.00 µg/mL | 1220.00 µg/mL | Bactericidal | 40.36 ± 0.67 | Anti-biofilm activity | Downregulated the expression of *hagA, hagB, hem, hmuR, ragA, ftn,* and *fimA-I* gene | [32] |
| *Syzygium aromaticum* | - | *P. gingivalis* ATCC 33277  *P. intermedia* ATCC 25611  *F. nucleatum* ATCC 10953  *S. sanguinis* ATCC 10556  S.gordonii ATCC 10558  *A. actinomycetemcomitans* ATCC 43717 | 100 µg/mL  100 µg/mL  100 µg/mL  400 µg/mL  100 µg/mL  800 µg/mL | 100 µg/mL  200 µg/mL  200 µg/mL  800 µg/mL  200 µg/mL  1600 µg/mL | Bactericidal  Bactericidal  Bactericidal  Bactericidal  Bactericidal  Bactericidal | -  -  -  -  -  - | -  -  -  -  -  - | -  -  -  -  -  - | [33] |
| *Nigella sativa* seeds | 3,7,7-Trimethylbicyclo[4.1.0]hept-3-ene-2,5-d  (22.05%), 1,3,8-p-Menthatriene (19.74%) | *P. gingivalis*  *T. forsythia*  *P. intermedia*  *A. actinomycetemcomitans* | 31.2 µg/mL  ＜31.2 µg/mL  31.2 µg/mL  ＜31.2 µg/mL | 15.6 µg/mL  15.6 µg/mL  15.6 µg/mL  15.6 µg/mL | Bactericidal  Bactericidal  Bactericidal  Bactericidal | 12.41 ± 0.41  13.05 ± 1.76  12.12 ± 0.61  15.11 ± 0.15 | -  -  -  - | -  -  -  - | [35] |
| *Mentha piperita*  (%[w/v]) | - | *P. gingivalis* strain W83  *T. denticola* strain CD-I  *P. intermedia*  *P. nigrescens* ATCC 25261  *F. nucleatum* FDC 364  *S. sanguinis*  *A. actinomycetemcomitans* ATCC 29524  *E. corrodens*  *A. viscosus* strain Ny-1 | 0.20 %  0.10 %  0.30 %  0.20 %  0.20 %  0.60 %  0.30 %  0.20 %  0.50 % | 0.43 %  -  -  -  -  ＞0.60 %  0.43 %  -  ＞0.60 % | Bactericidal  -  -  -  -  -  Bactericidal  -  - | -  -  -  -  -  -  -  -  - | -  -  -  -  -  -  -  -  - | -  -  -  -  -  -  -  -  - | [38] |
| *Rosmarinus officinalis*  (%[w/v]) | - | *P. gingivalis* strain W83  *T. denticola* strain CD-I  *P. intermedia*  *P. nigrescens* ATCC 25261  *F. nucleatum* FDC 364  *S. sanguinis*  *A. actinomycetemcomitans* ATCC 29524  *E. corrodens*  *A. viscosus* strain Ny-1 | not determined  not determined  not determined  not determined  not determined  ＞0.6 %  not determined  not determined  ＞0.6 % | -  -  -  -  -  -  -  -  - | -  -  -  -  -  -  -  -  - | -  -  -  -  -  -  -  -  - | -  -  -  -  -  -  -  -  - | -  -  -  -  -  -  -  -  - | [38] |
| *Salvia officinalis*  (%[w/v]) | - | *P. gingivalis* strain W83  *T. denticola* strain CD-I  *P. intermedia*  *P. nigrescens* ATCC 25261  *F. nucleatum* FDC 364  *S. sanguinis*  *A. actinomycetemcomitans* ATCC 29524  *E. corrodens*  *A. viscosus* strain Ny-1 | 0.06 %  0.20 %  not determined  0.06 %  0.08 %  0.60 %  0.20 %  0.10 %  0.50 % | 0.37 %  -  -  -  -  ＞0.60 %  0.57  -  ＞0.60 % | bacteriostatic  -  -  -  -  -  Bactericidal  -  - | -  -  -  -  -  -  -  -  - | -  -  -  -  -  -  -  -  - | -  -  -  -  -  -  -  -  - | [38] |
| *Ocimum tenuiflorum*  (%[w/v]) | - | *P. gingivalis* strain W83  *T. denticola* strain CD-I  *P. intermedia*  *P. nigrescens* ATCC 25261  *F. nucleatum* FDC 364  *S. sanguinis*  *A. actinomycetemcomitans* ATCC 29524  *E. corrodens*  *A. viscosus* strain Ny-1 | not determined  not determined  not determined  not determined  not determined  ＞0.6 %  not determined  not determined  ＞0.6 % | -  -  -  -  -  -  -  -  - | -  -  -  -  -  -  -  -  - | -  -  -  -  -  -  -  -  - | -  -  -  -  -  -  -  -  - | -  -  -  -  -  -  -  -  - | [38] |
| *Australian Melaleuca alternifolia*  (%[w/v]) | - | *P. gingivalis* strain W83  *T. denticola* strain CD-I  *P. intermedia*  *P. nigrescens* ATCC 25261  *F. nucleatum* FDC 364  *S. sanguinis*  *A. actinomycetemcomitans* ATCC 29524  *E. corrodens*  *A. viscosus* strain Ny-1 | 0.11 %  not determined  not determined  not determined  ＞0.6 %  not determined  0.11 %  not determined  0.6 % | ≥0.60 %  -  -  -  -  ＞0.60 %  ＞0.60 %  -  ＞0.60 % | -  -  -  -  -  -  -  -  - | -  -  -  -  -  -  -  -  - | -  -  -  -  -  -  -  -  - | -  -  -  -  -  -  -  -  - | [38] |
| Leaves of *Kielmeyera coriacea* Mart. & Zucc. | Germacrene D (24.2%), (E)-caryophyllene (15.5%), Bicyclogermacrene (11.6%) | *P. nigrescens* ATCC 33563  *S. sanguinis* ATCC 10556  *S. mitis* ATCC 49456  *A. naeslundii* ATCC 19039 | 200 µg/mL  >400 µg/mL  >400 µg/mL  >400 µg/mL | -  -  -  - | -  -  -  - | -  -  -  - | -  -  -  - | -  -  -  - | [41] |
| Inner bark of *Kielmeyera coriacea* Mart. & Zucc. | Alpha-copaene (14.9%), Alpha-E-bergamotene (13.0%), Beta-bisabolene (9.5%) | *P. nigrescens* ATCC 33563  *S. sanguinis* ATCC 10556  *S. mitis* ATCC 49456  *A. naeslundii* ATCC 19039 | 50 µg/mL  400 µg/mL  100 µg/mL  >400 µg/mL | -  -  -  - | -  -  -  - | -  -  -  - | -  -  -  - | -  -  -  - | [41] |
| Outer bark of *Kielmeyera coriacea* Mart. & Zucc. | Unidentified Oxygenated sesquiterpene (12.7%), Nonacosane (5.8%), Octacosane (5.0%) | *P. nigrescens* ATCC 33563  *S. sanguinis* ATCC 10556  *S. mitis* ATCC 49456  *A. naeslundii* ATCC 19039 | 100 µg/mL  100 µg/mL  100 µg/mL  400 µg/mL | -  -  -  - | -  -  -  - | -  -  -  - | -  -  -  - | -  -  -  - | [41] |
| Wood of *Kielmeyera coriacea* Mart. & Zucc. | Palmitic acid (16.2%), Nonacosane (9.7%), Octacosane (8.4%) | *P. nigrescens* ATCC 33563  *S. sanguinis* ATCC 10556  *S. mitis* ATCC 49456  *A. naeslundii* ATCC 19039 | 200 µg/mL  100 µg/mL  100 µg/mL  >400 µg/mL | -  -  -  - | -  -  -  - | -  -  -  - | -  -  -  - | -  -  -  - | [41] |
| *Stachys koelzii* | α-pinene (36.71%), 1,8-cineol (20.53%), trans-caryophyllene (12.34%) | *P. intermedia* ATCC 49046 | 100 µg/mL | 200 µg/mL | Bactericidal | - | Anti-biofilm activity | - | [42] |
| *Satureja hortensis* Linnaeus | Carvacrol (86.77%), Cymene (4.39%), α-Pinene (1.50%), Terpinol (1.02%) | *P. gingivalis* ATCC 33277  *P. gingivalis* AHN 24155  *P. gingivalis* AHN 24135  *T. forsythia* AHN 24212  *P. intermedia* ATCC 25611  *P. intermedia* AHN 8290  *P. nigrescens* ATCC 33563  *P. nigrescens* AHN 8293  *P. micra* ATCC 33270  *P. micra* AHC 15107  *P. micra* AHC 15154  *F. nucleatum* ATCC 25586  *F. nucleatum* AHN 9508  *A. actinomycetemcomitans* NTCC 9710  *A. actinomycetemcomitans* AHN 24195 | <0.125 μL/mL  <0.125 μL/mL  <0.125 μL/mL  <0.125 μL/mL  <0.125 μL/mL  <0.125 μL/mL  <0.125 μL/mL  <0.125 μL/mL  <0.125 μL/mL  <0.125 μL/mL  <0.125 μL/mL  <0.125 μL/mL  <0.125 μL/mL  <0.125 μL/mL  <0.125 μL/mL | -  -  -  -  -  -  -  -  -  -  -  -  -  -  - | -  -  -  -  -  -  -  -  -  -  -  -  -  -  - | -  -  -  -  -  -  -  -  -  -  -  -  -  -  - | -  -  -  -  -  -  -  Anti-biofilm activity  -  -  -  -  -  -  - | -  -  -  -  -  -  -  -  -  -  -  -  -  -  - | [43] |
| *Salvia fruticosa* Miller | 1,8-Cineol/eucalyptol (49.52%), Camphor (13.28%), β-pinene (7.19%), α-Pinene (5.82%) | *P. gingivalis* ATCC 33277  *P. gingivalis* AHN 24155  *P. gingivalis* AHN 24135  *T. forsythia* AHN 24212  *P. intermedia* ATCC 25611  *P. intermedia* AHN 8290  *P. nigrescens* ATCC 33563  *P. nigrescens* AHN 8293  *P. micra* ATCC 33270  *P. micra* AHC 15107  *P. micra* AHC 15154  *F. nucleatum* ATCC 25586  *F. nucleatum* AHN 9508  *A. actinomycetemcomitans* NTCC 9710  *A. actinomycetemcomitans* AHN 24195 | 8 μL/mL  8 μL/mL  8 μL/mL  8 μL/mL  8 μL/mL  8 μL/mL  8 μL/mL  2 μL/mL  8 μL/mL  8 μL/mL  8 μL/mL  8 μL/mL  8 μL/mL  8 μL/mL  8 μL/mL | -  -  -  -  -  -  -  -  -  -  -  -  -  -  - | -  -  -  -  -  -  -  -  -  -  -  -  -  -  - | -  -  -  -  -  -  -  -  -  -  -  -  -  -  - | -  -  -  -  -  -  -  -  -  -  -  -  -  -  - | -  -  -  -  -  -  -  -  -  -  -  -  -  -  - | [43] |
| *Lavandula stoechas* Linnaeus | Camphor (49.08%), Fenchone (27.66%), 1,8-Cineol/eucalyptol (13.94%) | *P. gingivalis* ATCC 33277  *P. gingivalis* AHN 24155  *P. gingivalis* AHN 24135  *T. forsythia* AHN 24212  *P. intermedia* ATCC 25611  *P. intermedia* AHN 8290  *P. nigrescens* ATCC 33563  *P. nigrescens* AHN 8293  *P. micra* ATCC 33270  *P. micra* AHC 15107  *P. micra* AHC 15154  *F. nucleatum* ATCC 25586  *F. nucleatum* AHN 9508  *A. actinomycetemcomitans* NTCC 9710  *A. actinomycetemcomitans* AHN 24195 | 4 μL/mL  4 μL/mL  4 μL/mL  4 μL/mL  4 μL/mL  4 μL/mL  4 μL/mL  4 μL/mL  4 μL/mL  4 μL/mL  4 μL/mL  4 μL/mL  4 μL/mL  4 μL/mL  4 μL/mL | -  -  -  -  -  -  -  -  -  -  -  -  -  -  - | -  -  -  -  -  -  -  -  -  -  -  -  -  -  - | -  -  -  -  -  -  -  -  -  -  -  -  -  -  - | -  -  -  -  -  -  -  -  -  -  -  -  -  -  - | -  -  -  -  -  -  -  -  -  -  -  -  -  -  - | [43] |
| *Myrtus communis* Linnaeus | 1,8-Cineol/eucalyptol (37.03%), α-Pinene (30.17%), Linalool (9.72%) | *P. gingivalis* ATCC 33277  *P. gingivalis* AHN 24155  *P. gingivalis* AHN 24135  *T. forsythia* AHN 24212  *P. intermedia* ATCC 25611  *P. intermedia* AHN 8290  *P. nigrescens* ATCC 33563  *P. nigrescens* AHN 8293  *P. micra* ATCC 33270  *P. micra* AHC 15107  *P. micra* AHC 15154  *F. nucleatum* ATCC 25586  *F. nucleatum* AHN 9508  *A. actinomycetemcomitans* NTCC 9710  *A. actinomycetemcomitans* AHN 24195 | 2 μL/mL  0.25 μL/mL  0.25 μL/mL  0.25 μL/mL  2 μL/mL  2 μL/mL  2 μL/mL  1 μL/mL  2 μL/mL  1 μL/mL  0.5 μL/mL  0.25 μL/mL  0.5 μL/mL  2 μL/mL  1 μL/mL | -  -  -  -  -  -  -  -  -  -  -  -  -  -  - | -  -  -  -  -  -  -  -  -  -  -  -  -  -  - | -  -  -  -  -  -  -  -  -  -  -  -  -  -  - | -  -  -  -  -  -  -  -  -  -  -  -  -  -  - | -  -  -  -  -  -  -  -  -  -  -  -  -  -  - | [43] |
| *Juniperus communis* Linnaeus | α-Pinene (79.45%), β-pinene (4.03%), 1,8-Cineol/eucalyptol (3.92%) | *P. gingivalis* ATCC 33277  *P. gingivalis* AHN 24155  *P. gingivalis* AHN 24135  *T. forsythia* AHN 24212  *P. intermedia* ATCC 25611  *P. intermedia* AHN 8290  *P. nigrescens* ATCC 33563  *P. nigrescens* AHN 8293  *P. micra* ATCC 33270  *P. micra* AHC 15107  *P. micra* AHC 15154  *F. nucleatum* ATCC 25586  *F. nucleatum* AHN 9508  *A. actinomycetemcomitans* NTCC 9710  *A. actinomycetemcomitans* AHN 24195 | 4 μL/mL  4 μL/mL  4 μL/mL  4 μL/mL  4 μL/mL  4 μL/mL  4 μL/mL  1 μL/mL  4 μL/mL  4 μL/mL  2 μL/mL  4 μL/mL  4 μL/mL  4 μL/mL  4 μL/mL | -  -  -  -  -  -  -  -  -  -  -  -  -  -  - | -  -  -  -  -  -  -  -  -  -  -  -  -  -  - | -  -  -  -  -  -  -  -  -  -  -  -  -  -  - | -  -  -  -  -  -  -  -  -  -  -  -  -  -  - | -  -  -  -  -  -  -  -  -  -  -  -  -  -  - | [43] |
| *Salvia officinalis* | α-thujone (25.778%), camphor (26.316%), 1,8-cineole (8.262%) | S 41.3 *P. oralis*  S 47 *S. mitis*  S 35.4 *S. intermedius*  S 41.1 *A. naeslundii*  S 41.2 *A. naeslundii*  S 114 *A. naeslundii* | -  45900 μg/mL  45900 μg/mL  22950 μg/mL  22950 μg/mL  - | -  -  -  -  -  - | -  -  -  -  -  - | -  -  -  -  -  - | -  -  -  -  -  - | -  -  -  -  -  - | [44] |
| *Satureja hortensis* Linnaeus | carvacrol (54.069%), γ-terpinene (26.749%), m-cymene (7.996%) | S 41.3 *P. oralis*  S 47 *S. mitis*  S 35.4 *S. intermedius*  S 41.1 *A. naeslundii*  S 41.2 *A. naeslundii*  S 114 *A. naeslundii* | 680 μg/mL  340 μg/mL  1360 μg/mL  1360 μg/mL  340 μg/mL  2720 μg/mL | -  -  -  -  -  - | -  -  -  -  -  - | -  -  -  -  -  - | -  -  -  -  -  - | -  -  -  -  -  - | [44] |
| *Anethum graveolens* | α-phellandrene (68.541%), β-phellandrene (9.431%) , carvacrol (8.625%) | S 41.3 *P. oralis*  S 47 *S. mitis*  S 35.4 *S. intermedius*  S 41.1 *A. naeslundii*  S 41.2 *A. naeslundii*  S 114 *A. naeslundii* | 1420 μg/mL  45500 μg/mL  710 μg/mL  45500 μg/mL  22750 μg/mL  22750 μg/mL | -  -  -  -  -  - | -  -  -  -  -  - | -  -  -  -  -  - | -  -  -  -  -  - | -  -  -  -  -  - | [44] |
| *Aloysia gratissima* (Aff & Hook).Tr | E-pinocamphone (16.07%), β-pinene (12.01%), Guaiol (8.53%) | *P. gingivalis* ATCC 33277  *F. nucleatum* ATCC 25586  *S. sanguinis* ATCC 10556  *S. mitis* ATCC 903 | 125 μg/mL  125 μg/mL  500 μg/mL  250 μg/mL | 125 μg/mL  250 μg/mL  1000 μg/mL  250 μg/mL | Bactericidal  Bactericidal  Bactericidal  Bactericidal | -  -  -  - | Anti-biofilm activity  Anti-biofilm activity  Anti-biofilm activity  Anti-biofilm activity | -  -  -  - | [51] |
| *Aloysia triphylla* (L’Hér.) Britton | - | *P. gingivalis* ATCC 33277  *F. nucleatum* ATCC 25586  *S. sanguinis* ATCC 10556  *S. mitis* ATCC 903 | 250 μg/mL  125 μg/mL  500 μg/mL  500 μg/mL | 250 μg/mL  250 μg/mL  1000 μg/mL  500 μg/mL | Bactericidal  Bactericidal  Bactericidal  Bactericidal | -  -  -  - | -  -  -  - | -  -  -  - | [51] |
| *Alpinia speciosa* (Pers.) Burtt & Smith | - | *P. gingivalis* ATCC 33277  *F. nucleatum* ATCC 25586  *S. sanguinis* ATCC 10556  *S. mitis* ATCC 903 | 125 μg/mL  125 μg/mL  500 μg/mL  500 μg/mL | 250 μg/mL  125 μg/mL  > 1000 μg/mL  - | Bactericidal  Bactericidal  -  - | -  -  -  - | -  -  -  - | -  -  -  - | [51] |
| *Baccharis dracunculifolia* DC | - | *P. gingivalis* ATCC 33277  *F. nucleatum* ATCC 25586  *S. sanguinis* ATCC 10556  *S. mitis* ATCC 903 | 125 μg/mL  125 μg/mL  500 μg/mL  250 μg/mL | 125 μg/mL  250 μg/mL  500 μg/mL  250 μg/mL | Bactericidal  Bactericidal  Bactericidal  Bactericidal | -  -  -  - | -  -  -  - | -  -  -  - | [51] |
| *Cinnamomun zeilanicus* Blume | - | *P. gingivalis* ATCC 33277  *F. nucleatum* ATCC 25586  *S. sanguinis* ATCC 10556  *S. mitis* ATCC 903 | 250 μg/mL  250 μg/mL  500 μg/mL  500 μg/mL | 250 μg/mL  250 μg/mL  1000 μg/mL  500 μg/mL | Bactericidal  Bactericidal  Bactericidal  Bactericidal | -  -  -  - | -  -  -  - | -  -  -  - | [51] |
| *Coriandrum sativum* L. | 1-decanol (33.91%), E-2-decen-1-ol (23.59%), 2-dodecen-1-ol (13.06%) | *P. gingivalis* ATCC 33277  *F. nucleatum* ATCC 25586  *S. sanguinis* ATCC 10556  *S. mitis* ATCC 903 | 125 μg/mL  15 μg/mL  250 μg/mL  62 μg/mL | 125 μg/mL  125 μg/mL  500 μg/mL  125 μg/mL | Bactericidal  Bactericidal  Bactericidal  Bactericidal | -  -  -  - | Anti-biofilm activity  Anti-biofilm activity  Anti-biofilm activity  Anti-biofilm activity | -  -  -  - | [51] |
| *Cymbopogon citratus* (DC) Stapf | - | *P. gingivalis* ATCC 33277  *F. nucleatum* ATCC 25586  *S. sanguinis* ATCC 10556  *S. mitis* ATCC 903 | 250 μg/mL  250 μg/mL  500 μg/mL  250 μg/mL | 250 μg/mL  250 μg/mL  > 1000 μg/mL  500 μg/mL | Bactericidal  Bactericidal  -  Bactericidal | -  -  -  - | -  -  -  - | -  -  -  - | [51] |
| *Cymbopogon. martini* (Roxb.) J.F. Watson | - | *P. gingivalis* ATCC 33277  *F. nucleatum* ATCC 25586  *S. sanguinis* ATCC 10556  *S. mitis* ATCC 903 | 250 μg/mL  125 μg/mL  500 μg/mL  250 μg/mL | 250 μg/mL  250 μg/mL  > 1000 μg/mL  250 μg/mL | Bactericidal  Bactericidal  -  Bactericidal | -  -  -  - | -  -  -  - | -  -  -  - | [51] |
| *Cymbopogon. winterianus* Jowitt | - | *P. gingivalis* ATCC 33277  *F. nucleatum* ATCC 25586  *S. sanguinis* ATCC 10556  *S. mitis* ATCC 903 | 250 μg/mL  125 μg/mL  500 μg/mL  250 μg/mL | 250 μg/mL  250 μg/mL  > 1000 μg/mL  500 μg/mL | Bactericidal  Bactericidal  -  Bactericidal | -  -  -  - | -  -  -  - | -  -  -  - | [51] |
| *Cyperus articulatus* L. | α-Pinene (5.72%), Mustakone (5.66%), α-bulnesene (5.02%) | *P. gingivalis* ATCC 33277  *F. nucleatum* ATCC 25586  *S. sanguinis* ATCC 10556  *S. mitis* ATCC 903 | 250 μg/mL  250 μg/mL  250 μg/mL  250 μg/mL | 250 μg/mL  250 μg/mL  500 μg/mL  500 μg/mL | Bactericidal  Bactericidal  Bactericidal  Bactericidal | -  -  -  - | Anti-biofilm activity  Anti-biofilm activity  Anti-biofilm activity  Anti-biofilm activity | Cell membrane disruption  Cell membrane disruption  Cell membrane disruption  Cell membrane disruption | [51] |
| *Elyonurus muticus* Spreng. | - | *P. gingivalis* ATCC 33277  *F. nucleatum* ATCC 25586  *S. sanguinis* ATCC 10556  *S. mitis* ATCC 903 | 250 μg/mL  250 μg/mL  500 μg/mL  500 μg/mL | 250 μg/mL  500 μg/mL  1000 μg/mL  - | Bactericidal  Bactericidal  Bactericidal  - | -  -  -  - | -  -  -  - | -  -  -  - | [51] |
| *Eugenia florida* DC. | - | *P. gingivalis* ATCC 33277  *F. nucleatum* ATCC 25586  *S. sanguinis* ATCC 10556  *S. mitis* ATCC 903 | > 1000 μg/mL  125 μg/mL  125 μg/mL  500 μg/mL | 125 μg/mL  250 μg/mL  250 μg/mL  500 μg/mL | Bactericidal  Bactericidal  Bactericidal  Bactericidal | -  -  -  - | -  -  -  - | -  -  -  - | [51] |
| *Eugenia uniflora* L | - | *P. gingivalis* ATCC 33277  *F. nucleatum* ATCC 25586  *S. sanguinis* ATCC 10556  *S. mitis* ATCC 903 | 250 μg/mL  125 μg/mL  500 μg/mL  500 μg/mL | 250 μg/mL  125 μg/mL  500 μg/mL  500 μg/mL | Bactericidal  Bactericidal  Bactericidal  Bactericidal | -  -  -  - | -  -  -  - | -  -  -  - | [51] |
| *Lippia alba* (Mill) N.E. Brown | - | *P. gingivalis* ATCC 33277  *F. nucleatum* ATCC 25586  *S. sanguinis* ATCC 10556  *S. mitis* ATCC 903 | 250 μg/mL  125 μg/mL  250 μg/mL  250 μg/mL | 250 μg/mL  125 μg/mL  1000 μg/mL  - | Bactericidal  Bactericidal  Bactericidal  - | -  -  -  - | -  -  -  - | -  -  -  - | [51] |
| *Lippia sidoides* Cham. | Thymol (65.76%), p-cymene (17.28%), α-caryophyllene (10.46%) | *P. gingivalis* ATCC 33277  *F. nucleatum* ATCC 25586  *S. sanguinis* ATCC 10556  *S. mitis* ATCC 903 | 250 μg/mL  125 μg/mL  125 μg/mL  250 μg/mL | 250 μg/mL  125 μg/mL  500 μg/mL  - | Bactericidal  Bactericidal  Bactericidal  - | -  -  -  - | Anti-biofilm activity  Anti-biofilm activity  Anti-biofilm activity  Anti-biofilm activity | -  -  -  - | [51] |
| *Mentha piperita* L. | - | *P. gingivalis* ATCC 33277  *F. nucleatum* ATCC 25586  *S. sanguinis* ATCC 10556  *S. mitis* ATCC 903 | 250 μg/mL  250 μg/mL  500 μg/mL  500 μg/mL | > 1000 μg/mL  250 μg/mL  500 μg/mL  500 μg/mL | -  Bactericidal  Bactericidal  Bactericidal | -  -  -  - | -  -  -  - | -  -  -  - | [51] |
| *Mikania glomerata* Spreng | Germacrene D(38.29%), Bicyclogermacrene (7.98%), α-caryophyllene (9.49%) | *P. gingivalis* ATCC 33277  *F. nucleatum* ATCC 25586  *S. sanguinis* ATCC 10556  *S. mitis* ATCC 903 | 500 μg/mL  250 μg/mL  62 μg/mL  125 μg/mL | > 1000 μg/mL  500 μg/mL  125 μg/mL  125 μg/mL | -  Bactericidal  Bactericidal  Bactericidal | -  -  -  - | Anti-biofilm activity  Anti-biofilm activity  Anti-biofilm activity  Anti-biofilm activity | -  -  -  - | [51] |
| *Siparuna guianenses* Aubl | - | *P. gingivalis* ATCC 33277  *F. nucleatum* ATCC 25586  *S. sanguinis* ATCC 10556  *S. mitis* ATCC 903 | 62 μg/mL  62 μg/mL  250 μg/mL  125 μg/mL | 125 μg/mL  250 μg/mL  1000 μg/mL  250 μg/mL | Bactericidal  Bactericidal  Bactericidal  Bactericidal | -  -  -  - | -  -  -  - | -  -  -  - | [51] |
| *Syzygium aromaticum* (L.) Merr. & L. M. Perry | - | *P. gingivalis* ATCC 33277  *F. nucleatum* ATCC 25586  *S. sanguinis* ATCC 10556  *S. mitis* ATCC 903 | 250 μg/mL  250 μg/mL  500 μg/mL  500 μg/mL | 250 μg/mL  250 μg/mL  1000 μg/mL  500 μg/mL | Bactericidal  Bactericidal  Bactericidal  Bactericidal | -  -  -  - | -  -  -  - | -  -  -  - | [51] |
| *Ziziphus joazeiro* mart | - | *P. gingivalis* ATCC 33277  *F. nucleatum* ATCC 25586  *S. sanguinis* ATCC 10556  *S. mitis* ATCC 903 | 250 μg/mL  250 μg/mL  500 μg/mL  500 μg/mL | 250 μg/mL  500 μg/mL  1000 μg/mL  500 μg/mL | Bactericidal  Bactericidal  Bactericidal  Bactericidal | -  -  -  - | -  -  -  - | -  -  -  - | [51] |
| *Rhododendron groenlandicum*  (%[v/v]) | sabinene (11.93%), β-selinene (10.95%), Germacrene B (9.76%) | *F. nucleatum* ATCC 25586  *F. nucleatum* ATCC 10953  *F. nucleatum* ATCC 49256 | 0.25 %  0.25 %  0.25 % | 0.5 %  0.5 %  0.5 % | Bactericidal  Bactericidal  Bactericidal | -  -  - | Reduce VSCs production and no Anti-biofilm activity  -  - | Cell membrane disruption  -  - | [52] |
| *Mentha piperita*  (%[v/v]) | menthol (42.3%), menthone (24.7%), Menthofurane (7.1%) | *F. nucleatum* ATCC 25586  *F. nucleatum* ATCC 10953  *F. nucleatum* ATCC 49256 | 0.25 %  0.25 %  0.5 % | 0.5 %  0.5 %  1 % | Bactericidal  Bactericidal  Bactericidal | -  -  - | Reduce VSCs production and no Anti-biofilm activity  -  - | Cell membrane disruption  -  - | [52] |
| *Satureja montana*  (%[v/v]) | carvacrol (43.8%), p-cymene (14.3%), γ-Terpinene (12.66%) | *F. nucleatum* ATCC 25586  *F. nucleatum* ATCC 10953  *F. nucleatum* ATCC 49256 | 0.031 %  0.031 %  0.063 % | 0.031 %  0.031 %  0.063 % | Bactericidal  Bactericidal  Bactericidal | -  -  - | Reduce VSCs production and no Anti-biofilm activity  -  - | Cell membrane disruption  -  - | [52] |
| *Asarum heterotropoides* var. Mandshuricum  (%[v/v]) | Methyl eugenol (45.95%), Safrole(17.48%) | *P. gingivalis* JCM 12257  *P. intermedia* JCM 12248  *F. nucleatum* JCM 11024 | 0.005 %  0.04 %  0.01 % | 0.005 %  0.08 %  0.02 % | Bactericidal  Bactericidal  Bactericidal | -  -  - | -  -  -  - | -  -  -  - | [53] |
| *Lavandula angustifolia* | Camphor (33.26%), Eucalyptol (17.06%), Linalyl acetate (12.18%) | *F. nucleatum* PK1594 | 4 µL/mL | - | - | - | Reduce VSCs production | Cell membrane disruption | [55] |
| *Satureja hortensis* L.  (%[v/v]) | - | *S. sanguinis* PTCC1449 | 1.5625 % | - | - | 28.83 ±1.89 (50 %)  22 ± 1 (25 %)  18.6 ± 0.57 (12.5 %)  17 ± 1 (6.25%)  11.3 ± 1.15 (3.125 %) | - | - | [57] |
| fresh leaf of *Psidium guajava* L. | β-Caryophyllene（16.1%, α-Humulene（11.9%), Aromadendrene oxide（14.7%） | *S. sanguinis* ATCC 10556  *S. mitis* ATCC 49452 | 400 µg/mL  200 µg/mL | -  - | -  - | -  - | -  - | -  - | [58] |
| *Scheelea phalerata* (dry seasons) | Phytol (36.7%), Nonadecane (9.7%), Linolenic acid (9.1%) | *P. gingivalis* ATCC 33277  *F. nucleatum* ATCC 25586  *S. sanguinis* ATCC 10556  *S. mitis* ATCC 49456  *A. actinomycetemcomitans* ATCC 43717  *A. naeslundii*c ATCC 19039 | 400 µg/mL  400 µg/mL  200 µg/mL  400 µg/mL  400 µg/mL  400 µg/mL | -  -  -  -  -  - | -  -  -  -  -  - | -  -  -  -  -  - | -  -  -  -  -  - | -  -  -  -  -  - | [59] |
| *Scheelea phalerata*  (rainy seasons) | Phytol (26.1%), Palmitic acid (18.7%), Hexan-1-ol (15.6%) | *P. gingivalis* ATCC 33277  *F. nucleatum* ATCC 25586  *S. sanguinis* ATCC 10556  *S. mitis* ATCC 49456  *A. actinomycetemcomitans* ATCC 43717  *A. naeslundii*c ATCC 19039 | ＞400 µg/mL  ＞400 µg/mL  ＞400 µg/mL  ＞400 µg/mL  ＞400 µg/mL  ＞400 µg/mL | -  -  -  -  -  - | -  -  -  -  -  - | -  -  -  -  -  - | -  -  -  -  -  - | -  -  -  -  -  - | [59] |
| *Piper marginatum* | 3,4-(methylenedioxy propiophenone (11.3%), germacrene-D (10.8%) | *S. sanguinis* ATCC 10556  *S. mitis* ATCC 49456 | 225 µg/mL  75 µg/mL | -  - | -  - | -  - | -  - | -  - | [60] |
| *Piper callosum* | α-pinene (19.2%), β-pinene (14.3%), methyl eugenol (6.5%) | *S. sanguinis* ATCC 10556  *S. mitis* ATCC 49456 | 1000 µg/mL  500 µg/mL | -  - | -  - | -  - | -  - | -  - | [60] |
| *Peperomia pellucida* | Dillapiole(40.6%), viridiflorol (15.1%) | *S. sanguinis* ATCC 10556  *S. mitis* ATCC 49456 | 250 µg/mL  125 µg/mL | -  - | -  - | -  - | -  - | -  - | [60] |
| seeds of *Ferula assa-foetida* | Alpha.-D-Xylofuranoside, methyl  2,5-di-O-methyl- (30.2%), E-1-propenyl sec-butyl disulfide (13.13%) | *S. sanguinis* PTCC1449 | - | - | - | 6.94 ± 0.11 (2.5 µg/mL )  7.93 ± 0.30 (5 µg/mL )  9.90 ± 0.2 (10 µg/mL )  11.94 ± 0.20 (20 µg/mL ) | - | - | [61] |
| oleo-gum-resin of *Ferula assa-foetida* | E-1-propenyl sec-butyl disulfide (36.15%), Z-1-propenyl sec-butyl disulfide (27.93%) | *S. sanguinis* PTCC1449 | - | - | - | 7.07 ± 0.98 (2.5 µg/mL )  12.0 ± 0.00 (5 µg/mL )  12.17 ± 0.15 (10 µg/mL )  13.0 ± 0.00 (20 µg/mL ) | - | - | [61] |
| *Citrus aurantifolia* leaves | limonene (32.7%), linalool (20.1%), citronellal (14.5%) | *S. sanguinis* ATCC 10556  *S. mitis* ATCC 49456 | 200 µg/mL  200 µg/mL | -  - | -  - | -  - | -  - | -  - | [62] |
| *Citrus aurantifolia* fruit peel | limonene (77.5%), Myrcene (4.4%), Linalool (3.5%) | *S. sanguinis* ATCC 10556  *S. mitis* ATCC 49456 | 100 µg/mL  100 µg/mL | -  - | -  - | -  - | -  - | -  - | [62] |
| *Citrus medica* | Limonene (92.31%) | *S. sanguinis* PTCC1449 | 200000 µg/mL | - | - | 15 | - | - | [64] |
| *Pimpinella anisum* | (E)-Anethole (89.03%) | *S. sanguinis* PTCC1449 | 1820 µg/mL | 1820 µg/mL | Bactericidal | 15 | - | - | [64] |
| *Artemisia dracunculus* | Methyl chavicol (Estragole) (75.77%) | *S. sanguinis* PTCC1449 | 28440 µg/mL | 56880 µg/mL | Bactericidal | 16 | - | - | [64] |
| *Cymbopogon martinii* | Geraniol (83.5%), Geranyl acetate (8.4%) | *S. sanguinis*  *S. mitis* | 250 µg/mL  250 µg/mL | ＞2000 µg/mL  ＞2000 µg/mL | bacteriostatic  bacteriostatic | -  - | -  Anti-biofilm activity | -  - | [65] |
| *Thymus zygis* | Thymol (35%), p-Cymene (24.1%) | *S. sanguinis*  *S. mitis* | 1000 µg/mL  1000 µg/mL | 2000 µg/mL  2000 µg/mL | Bactericidal  Bactericidal | -  - | -  Anti-biofilm activity | -  - | [65] |
| *Thymus vulgaris* | Thymol (25.22%), Carvacrol (23.78%) | *S. sanguinis* ATCC 10556  *S. mitis* ATCC 9811  *S. gordonii* ATCC 10558  *A. naeslundii* ATCC 4356 | 1.32 µg/mL  1.32 µg/mL  1.32 µg/mL  0.32 µg/mL | 2.61 µg/mL  2.61 µg/mL  5.23 µg/mL  2.61 µg/mL | Bactericidal  Bactericidal  Bactericidal  bacteriostatic | 21  36  18  24 | -  -  -  - | -  -  -  - | [66] |
| *Hyptis spicigera* | α-Pinene (50.78%), 1,8-cineole (20.31%), β-pinene (18.30%) | *S. sanguinis* ATCC 10556  *S. mitis* ATCC 9811  *S. gordonii* ATCC 10558  *A. naeslundii* ATCC 4356 | 2.61 µg/mL  10.54 µg/mL  2.61 µg/mL  1.32 µg/mL | 5.23 µg/mL  5.23 µg/mL  10.54 µg/mL  2.61 µg/mL | Bactericidal  Bactericidal  bacteriostatic  Bactericidal | 12  19  11  19 | -  -  -  - | -  -  -  - | [66]  [120] |
| *Cymbopogon citratus* | trans-citral (geranial) (46.6%), cis-citral (neral) (34.1%) | *S. sanguinis* ATCC 10556  *S. mitis* ATCC 9811  *S. gordonii* ATCC 10558  *A. naeslundii* ATCC 19039 | 2610 µg/mL  2610 µg/mL  1320 µg/mL  1320 µg/mL | 10540 µg/mL  5230 µg/mL  5230 µg/mL  5230 µg/mL | bacteriostatic  Bactericidal  Bactericidal  Bactericidal | 10  19  10  16 | -  -  -  - | -  -  -  - | [70] |
| *Curcuma aeruginosa* | Alloaromadendrene (25.15%), Curzerene (22.15%) | *S. sanguinis* ATCC 10556  *S. mitis* ATCC 49456 | -  - | -  - | -  - | 14.54 ± 0.29  12.35 ± 0.50 | -  - | -  - | [71] |
| *Curcuma mangga* | β-Myrcene (79.77%), β-Pinene (11.18%) | *S. sanguinis* ATCC 10556  *S. mitis* ATCC 49456 | -  - | -  - | -  - | 13.22 ± 0.53  17.78 ± 1.17 | -  - | -  - | [71] |
| *Curcuma xanthorrhiza* | β-Curcumene (34.90%), α-Curcumene (24.54%), Germacrone (9.13%) | *S. sanguinis* ATCC 10556  *S. mitis* ATCC 49456 | -  - | -  - | -  - | 15.04 ± 3.05  19.50 ± 2.22 | -  - | -  - | [71] |
| *Kaempferia galanga* | Ethyl-cinnamate (40.14%), Pentadecane (15.06%) | *S. sanguinis* ATCC 10556  *S. mitis* ATCC 49456 | -  - | -  - | -  - | 7.18 ± 0.42  8.56 ± 1.02 | -  - | -  - | [71] |
| *Nepeta Cataria* at the vegetative stage | 4a-α,7-α,7a-β-Nepetalactone (55%)  4a-α,7-β,7a-α-Nepetalactone (30.06%) | *S. sanguinis* ATCC 10556 | 1 µL/mL | 2 µL/mL | Bactericidal | - | - | - | [72] |
| *Nepeta Cataria* at the floral budding stage | 4a-α,7-α,7a-β-Nepetalactone (58%)  4a-α,7-β,7a-α-Nepetalactone (31.1%) | *S. sanguinis* ATCC 10556 | 1 µL/mL | 2 µL/mL | Bactericidal | - | - | - | [72] |
| *Nepeta Cataria* at the full flowering stage | 4a-α,7-α,7a-β-Nepetalactone (55.03%)  4a-α,7-β,7a-α-Nepetalactone (31.2%) | *S. sanguinis* ATCC 10556 | 1 µL/mL | 2 µL/mL | 三菌 | - | - | - | [72] |
| *Cimbopogon winterianus*  (%[v/v]) | - | *S. intermedius* Clinical Isolate | 2.5 % | - | - | - | no Anti-biofilm activity | - | [73] |
| *Origanum syriacum*  (%[v/v]) | 无 | *S. intermedius* Clinical Isolate | 1.25 % | - | - | - | no Anti-biofilm activity | - | [73] |
| *Tunisian Nigella sativa* seeds | p-cymene (49.48%) , α-thujene (18.93%), α-pinene (5.44%), β-pinene (4.31%) , γ-terpinene (3.69%), Limonene (2.89%), α-terpinene (2.47%), Thymoquinone（0.79 %） | *S. sanguinis* B747  *S. sanguinis* B535  *S. oralis* B634  *S. oralis* B154  *S. oralis* B736  *S. mitis* B116  *S. mitis* B627  *S. mitis* B546  *S. mitis* B120  *S. mitis* B576 | 8500 µg/mL  8500 µg/mL  2130 µg/mL  4250 µg/mL  4250 µg/mL  4250 µg/mL  2130 µg/mL  2130 µg/mL  4250 µg/mL  2130 µg/mL | -  -  -  -  -  -  -  -  -  - | -  -  -  -  -  -  -  -  -  - | 10.5 ± 0.707  11.5 ± 0.707  13.5 ± 0.707  15.5 ± 0.707  14.5 ± 0.707  11.5 ± 0.707  15.5 ± 0.707  14.5 ± 0.707  11.5 ± 0.707  14.5 ± 0.707 | -  -  -  -  -  -  -  -  -  - | -  -  -  -  -  -  -  -  -  - | [74] |
| *Melampodium divaricatum* (Rich.) DC. | (E)-caryophyllene (56.0%), germacrene D (12.7%) | *S. sanguinis* ATCC 10556  *S. mitis* ATCC 49456 | 300 µg/mL  18 µg/mL | -  - | -  - | -  - | -  - | -  - | [76] |
| *Pistacia vera* L. | α-Pinene (91.25%), β-Pinene (1.12%) | *S. sanguinis* ATCC 10556  *S. oralis* ATCC 10557  *S. intermedius* ATCC 27335 | 512 µg/mL  1024 µg/mL  256 µg/mL | 512 µg/mL  1024 µg/mL  256 µg/mL | Bactericidal  Bactericidal  Bactericidal | -  -  - | -  -  - | -  -  - | [77] |
| *Plectranthus neochilus* | trans-caryophyllene (29.8%), α-pinene (14.1%) | *S. sanguinis* ATCC 10556  *S. mitis* ATCC 49456 | 62.5 µg/mL  31.3 µg/mL | -  - | -  - | -  - | -  - | -  - | [78] |
| *Rosmarinus officinalis* | Camphor (18.91%), Verbenone (11.32%), α-Pinene (9.61%) | *S. sanguinis* ATCC 10556  *S. mitis* ATCC 49456 | > 2000 μg/mL  > 2000 μg/mL | -  - | -  - | -  - | -  - | -  - | [79] |
| *Thymus vulgaris* | - | *P. gingivalis* Clinical Isolate  *A. actinomycetemcomitans* Clinical Isolate | 32 μg/mL  32 μg/mL | -  - | -  - | 8.2 ± 0.4  10.9 ± 0.9 | -  - | -  - | [82] |
| *Origanum vulgare* | Carvacrol (32.36%), α-terpineol (16.70%), p-cymene (16.25%) | *A. actinomycetemcomitans* | 0.05-1.51 µg/mL | 0.09-2.01 µg/mL | Bactericidal | 37 ± 1.73-69.66 ± 0.57 | -  - | -  - | [83] |
| *Melaleuca alternifolia* | Terpinen-4-ol (38.7%), γ-Terpinene (23.9%), α-Terpinene (14.8%) | *S. sanguinis* ATCC 10556  *S. oralis* ATCC 10557  *A. actinomycetemcomitans* ATCC 29522 | 40000 µg/mL  40000 µg/mL  16700 µg/mL | -  -  - | -  -  - | -  -  - | -  -  - | -  -  - | [84] |
| *Cryptomeria japonica* | elemol (11.17%), terpinen-4-ol (9.77%), sabinene (8.86%) | *P. gingivalis* ATCC 33277  *P. intermedia* ATCC 25611  *F. nucleatum* ATCC 10953  *S. sanguinis* ATCC 10556  S.gordonii ATCC 10558  *A. actinomycetemcomitans* ATCC 43717 | 25 µg/mL  50 µg/mL  50 µg/mL  100 µg/mL  25 µg/mL  400 µg/mL | 50 µg/mL  100 µg/mL  100 µg/mL  100 µg/mL  50 µg/mL  800 µg/mL | Bactericidal  Bactericidal  Bactericidal  Bactericidal  Bactericidal  Bactericidal | -  -  -  -  -  - | -  -  -  -  -  - | -  -  -  -  -  - | [85] |
| *Psidium cattleianum* Sabine (Myrtaceae) Fresh Leaves | Viridiflorol (17.9%), β-caryophyllene (11.8%), 1,8-Cineole (10.8%) | *P. gingivalis* ATCC 33277  *P. nigrescens* ATCC 33563  *F. nucleatum* ATCC 25586  *A. actinomycetemcomitans* ATCC 43717  *A. naeslundii* ATCC 19039 | 20 µg/mL  62.5 µg/mL  12.5 µg/mL  6.25 µg/mL  50 µg/mL | -  -  -  -  - | -  -  -  -  - | -  -  -  -  - | -  -  -  -  - | -  -  -  -  - | [86] |
| *Pimpinella anisum*  (%[v/v]) | 无 | *A. actinomycetemcomitans* JP2NOV99  *A. naeslundii* PTCC 1201 | 9.76 %  4.88 % | 9.76 %  9.76 % | Bactericidal  Bactericidal | 18.5 ± 1.29  42 ± 1.63 | -  - | -  - | [94] |
| *Myristica fragrans* | sabinene (36.6%), alpha-Pinene (14.4%), beta.‑Pinene (10.3%) | *P. gingivalis* ATCC 33277  *P. intermedia* ATCC 25611  *A. viscosus* ATCC 10048 | 8 µg/mL  4 µg/mL  8 µg/mL | -  -  - | -  -  - | -  -  - | -  -  - | -  -  - | [95] |
| *Lavandula angustifolia* (through hydrodistillation) | Linalool (30.50±0.18%), Linalyl acetate (22.80±0.51%), Lavandulyl acetate (11.20±0.76%) | *A. viscosus* CCTCC AB 99001 | 250 µg/mL | - | - | 9.3 ± 0.6 | - | - | [96] |
| Mixtures *(*Essential oils of *Salvia officinalis, Mentha piperita, Lippia citriodora* and aqueous extract of *Matricaria chamomilla,*  *Echinacea purpurea )* | - | *S. sanguinis* PTCC1449  *E. corrodens* PTCC1391  A. viscousus PTCC1202 | 0.4 µg/mL  25 µg/mL  0.8 µg/mL | 6 µg/mL  25 µg/mL  12 µg/mL | bacteriostatic  Bactericidal  bacteriostatic | -  -  - | Anti-biofilm activity  Anti-biofilm activity  Anti-biofilm activity | -  -  - | [89] |
| *Cinnamomum camphora cineoliferum* | 1,8-cineol (59.5%) , sabinene (15.3%), α-terpineol (6.1%) | *Prevotella denticola*  *Prevotella buccae*  *Prevotella oralis*  *F. nucleatum* ATCC 25586  *S. sanguinis* Clinical Isolate 1  *S. sanguinis* Clinical Isolate 3  *S. sanguinis* Clinical Isolate 4  *S. sanguinis* Clinical Isolate 5  *S. sanguinis* ATCC 10556  *S. mitis*  *V. parvula* ATCC 10790 | 16150 μg/mL  5000 μg/mL  4550 μg/mL  1250 μg/mL  11150 μg/mL  22500 μg/mL  22500 μg/mL  15950 μg/mL  4650 μg/mL  16400 μg/mL  4550 μg/mL | 32300 μg/mL  10000 μg/mL  9100 μg/mL  2550 μg/mL  15000 μg/mL  37500 μg/mL  37500 μg/mL  31900 μg/mL  9350 μg/mL  32800 μg/mL  9100 μg/mL | Bactericidal  Bactericidal  Bactericidal  Bactericidal  Bactericidal  Bactericidal  Bactericidal  Bactericidal  Bactericidal  Bactericidal  Bactericidal | -  -  -  -  -  -  -  -  -  -  - | -  -  -  -  -  -  -  -  -  -  - | -  -  -  -  -  -  -  -  -  -  - | [91] |
| *Melaleuca ericifolia* | linalool (49.1%), 1,8-cineol 桉叶油醇 (26%) , α-pinene (12.0%) | *Prevotella denticola*  *Prevotella buccae*  *Prevotella oralis*  *F. nucleatum* ATCC 25586  *S. sanguinis* Clinical Isolate 1  *S. sanguinis* Clinical Isolate 3  *S. sanguinis* Clinical Isolate 4  *S. sanguinis* Clinical Isolate 5  *S. sanguinis* ATCC 10556  *S. mitis*  *V. parvula* ATCC 10790 | 2500 μg/mL  5300 μg/mL  5300 μg/mL  650 μg/mL  13650 μg/mL  23350 μg/mL  4350 μg/mL  23350 μg/mL  10150 μg/mL  11700 μg/mL  650 μg/mL | 5000 μg/mL  10600 μg/mL  10600 μg/mL  1350 μg/mL  23350 μg/mL  38900 μg/mL  8750 μg/mL  38950 μg/mL  14000 μg/mL  23350 μg/mL  1350 μg/mL | Bactericidal  Bactericidal  Bactericidal  Bactericidal  Bactericidal  Bactericidal  Bactericidal  Bactericidal  Bactericidal  Bactericidal  Bactericidal | -  -  -  -  -  -  -  -  -  -  - | -  -  -  -  -  -  -  -  -  -  - | -  -  -  -  -  -  -  -  -  -  - | [91] |
| *Lavandula angustifolia* (through microwave-assisted hydrodistillation ) | Linalool (32.90±0.83%), Linalyl acetate (27.00±0.15%), Lavandulyl acetate (10.90±0.14%) | *A. viscosus* CCTCC AB 99001 | 125 µg/mL | - | - | 10.2 ± 0.3 | - | - | [96] |
| *Sal. fruticosa* M. | - | *F. nucleatum* ATCC 25586 | - | - | - | - | - | Cell membrane disruption | [99] |
| *Satureja hortensis* L. | - | *F. nucleatum* ATCC 25586 | - | - | - | - | - | Cell membrane disruption | [99] |
| *Cinnamomum cassia* | cinnamaldehyde (57.971%), eugenol (19.188%), linalool (4.563%) | *P. gingivalis* ATCC 33177 | 6.25 µg/mL | - | - | - | Anti-biofilm activity | Cell membrane disruption | [100] |
| *Elsholtzia ciliate* | Carvacrol (26.2465%), p-Cymene (21.5087%), Phellandrene (20.0729%) | *P. gingivalis* ATCC 33277  *F. nucleatum* ATCC 25586 | 0.1 µL/mL  0.1 µL/mL | 0.2 µL/mL  0.2 µL/mL | Bactericidal  Bactericidal | -  - | Reduce VSCs production、Anti-biofilm activity | Cell membrane disruption  Cell membrane disruption | [101] |
| *Nigella sativa* | - | *F. nucleatum* ATCC 25586 | 63 µg/mL | - | - | - | - | bacteriostatic of L-methionine-γ-lyase | [104] |
| *Syzygium aromaticum* leaves | eugenol (90.84%), β-caryophyllene (5.05%) | *P. gingivalis* ATCC 33277 | 6.25 µg/mL | 25 µg/mL | Bactericidal | - | - | Eugenol downregulated the expression of *fimA, hagA, hagB, rgpA, rgpB,* and *kgp* gene | [108] |

MIC: minimum inhibitory concentration;

MBC: minimum bactericidal concentration;

DIZ: diameter of bacteriostatic zone;

The ratio of MBC/MIC ≤ 4 suggests bactericidal activity, while the ratio > 4 indicates bacteriostatic activity.
